# Drought facilitated the westward expansion of the Mongol Empire in the 1230s

**DOI:** 10.1016/j.fmre.2025.08.010

**Published:** 2025-09-03

**Authors:** Weipeng Yue, Feng Chen, Olga Solomina, Jan Esper, Nicole K. Davi, Ulf Büntgen, Shijie Wang, Vladimir Matskovsky, Caroline Leland, Leonid Agafonov, Max C.A. Torbenson, Magdalena Opała-Owczarek, Mao Hu, Marina Gurskaya, Zulfiyor Bakhtiyorov, Xiaoen Zhao, Yang Xu, Heli Zhang, Youping Chen, Fahu Chen

**Affiliations:** a Yunnan Key Laboratory of International Rivers and Transboundary Eco-Security, Institute of International Rivers and Eco-Security, Yunnan University, Kunming 650500, China; b Department of Geography, Johannes Gutenberg University, Mainz 55122, Germany; c Southwest United Graduate School, Kunming 650500, China; d Key Laboratory of Tree-ring Physical and Chemical Research of the China Meteorological Administration/Xinjiang Laboratory of Tree-ring Ecology, China Meteorological Administration, Institute of Desert Meteorology, Urumqi 830002, China; e Institute of Geography, Russian Academy of Sciences, Moscow 119017, Russia; f National Research University Higher School of Economics, Moscow 109028, Russia; g Global Change Research Institute, Czech Academy of Sciences, Brno 60300, Czech Republic; h Department of Environmental Science, William Paterson University, Wayne 07470, USA; i Tree-Ring Lab, Lamont-Doherty Earth Observatory, Columbia University, NY 10964, USA; j Department of Geography, University of Cambridge, Cambridge CB2 3EN, UK; k Department of Geography, Faculty of Science, Masaryk University, Brno 61137, Czech Republic; l Laboratory of Dendrochronology, Russian Academy of Sciences, Institute of Plant and Animal Ecology, Yekaterinburg 620144, Russia; m Institute of Earth Sciences, Faculty of Natural Sciences, University of Silesia in Katowice, Katowice 41-200, Poland; n Xinjiang Institute of Ecology and Geography, Chinese Academy of Sciences, Urumqi 830011, China; o Khujand Science Center, National Academy of Sciences of the Republic of Tajikistan, Khujand 735714, Tajikistan; p ALPHA, State Key Laboratory of Tibetan Plateau Earth System, Environment and Resources (TPESER), Institute of Tibetan Plateau Research (ITPCAS), Chinese Academy of Sciences (CAS), Beijing 100101, China; q College of Resources and Environment, University of Chinese Academy of Sciences, Beijing 100049, China; r MOE Key Laboratory of Western China’s Environmental System, Lanzhou University, Lanzhou 730000, China

**Keywords:** Mongol Empire, Western expedition, East European Plain, Water balance, Summer North Atlantic Oscillation, Drought Risk

## Abstract

The Mongol Empire’s Western Expedition stands as the most impactful historical event shaping the historical trajectory of the Eurasian continent in the second millennium. However, the climatic spatiotemporal patterns during the Mongol Empire’s Western Expedition have remained inadequately elucidated. Here, we reconstruct changes in summer water balance on the East European Plain between 943 and 2019 CE. Integrating tree-ring hydroclimatic sequences from Central Europe, Central Asia, and East Asia, we comprehensively delineate the climatic backdrop along the empire’s western expansion route, demonstrating the pivotal role of climate in driving the Mongol Empire’s expansion. We find that the persistent arid grassland environment along the main routes of the Mongol Empire’s westward expansion (1227–1241 CE) provided climatic advantages for their expansion, enhancing the military effectiveness of Mongol cavalry and driving the rapid conquest of the Eurasian continent. We identify the summer North Atlantic Oscillation as a primary driver of East European Plain hydroclimatic variability and highlight increased drought risk under future warming scenarios. By integrating long-term climate variability with sociohistorical dynamics, this study proposes a framework for understanding the mechanisms underpinning climate-human interactions.

## Highlights

• Reconstructed summer water balance on the East European Plain from 943 to 2019 CE using tree-ring data, providing the climatic context during the Mongol Empire’s western expedition.
• Persistent arid grassland environments facilitated the Mongol Empire’s rapid conquest of the Eurasian continent.
• Identified the summer North Atlantic Oscillation as the dominant driver of hydroclimatic variability in the East European Plain, and emphasized increased future drought risk under warming scenarios.

## Graphical abstract

Climatic and hydrological background of the westward expansion of the Mongolian Empire. (a) Changes in the warm-season water balance of Mongolia reconstructed. (b) Changes in the runoff of Central Asia reconstructed. (c) Precipitation variability in western Central Asia (Pamir-Alai) for the past 1100 years. (d) Precipitation variability over the past nine centuries in Central Asia (Altai Mountains). (e) Changes in the water balance in the EEP (this study). (f) Central European water balance reconstruction from tree-ring stable carbon and oxygen isotopes (δ_13_C and δ_18_O). The above water balance and runoff results reconstructed from tree ring data are only shown for 1100–1399 CE. Change points are used to define dry/wet or high/low runoff shifts. (g) Comparison of boxplots of dry/wet or high/low runoff for 10 years before and after the detected change point, corresponding to the years 1210, 1239, 1210, 1219, 1227, and 1235. (h), (i), (j), (k) respectively show the spatial distribution of PDSI in the first 10 years minus the last 10 years of the main change points (the years 1210, 1219, 1227, 1235). Data are from the Paleo Hydrodynamics Data Assimilation product (PHYDA) and the approximate spatial locations (red capital letters) and time context of the five main conquered objects on the Mongol Empire’s Western Expedition route are also provided in the figure.

**Figure:**
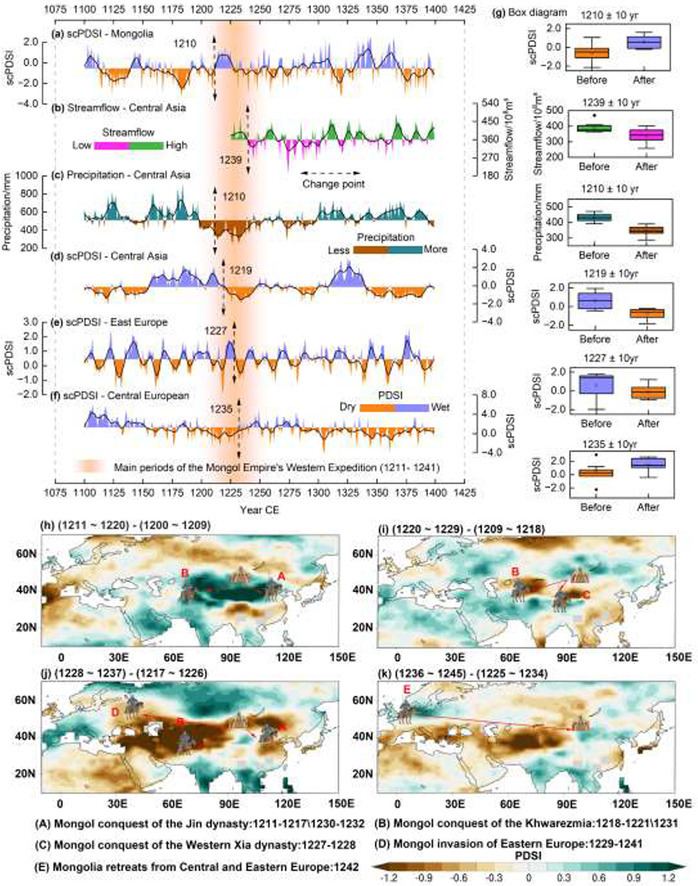

## 1 Introduction

**Fig. 1:**
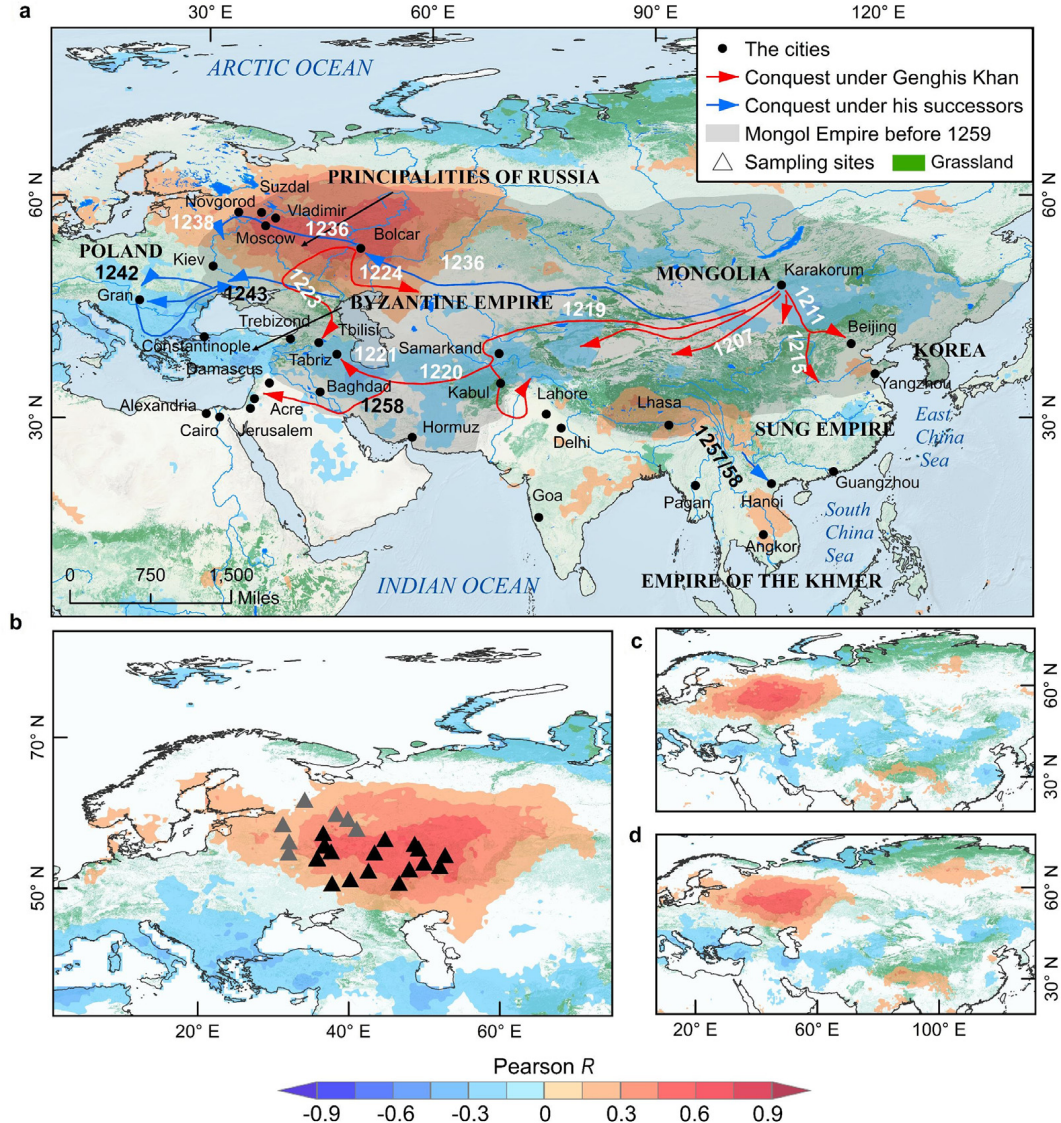
**Map of the study area.** (a) The land area ruled by the Mongol Empire before 1259, with red and blue arrows indicating the principal directions of advance of Genghis Khan and his successors in their conquest of Eurasia, while black dots represent cities [76]. Correlation results of the reconstructed scPDSI and CRU grid drought datasets based on original (b), detrended (c), and first-order differences (d) (1901–2019). The triangles represent sampling sites (black: living trees, grey: archaeological samples). All regions in the spatial correlation field that fail the significance test (*p* < 0.05) are masked.

**Fig. 2:**
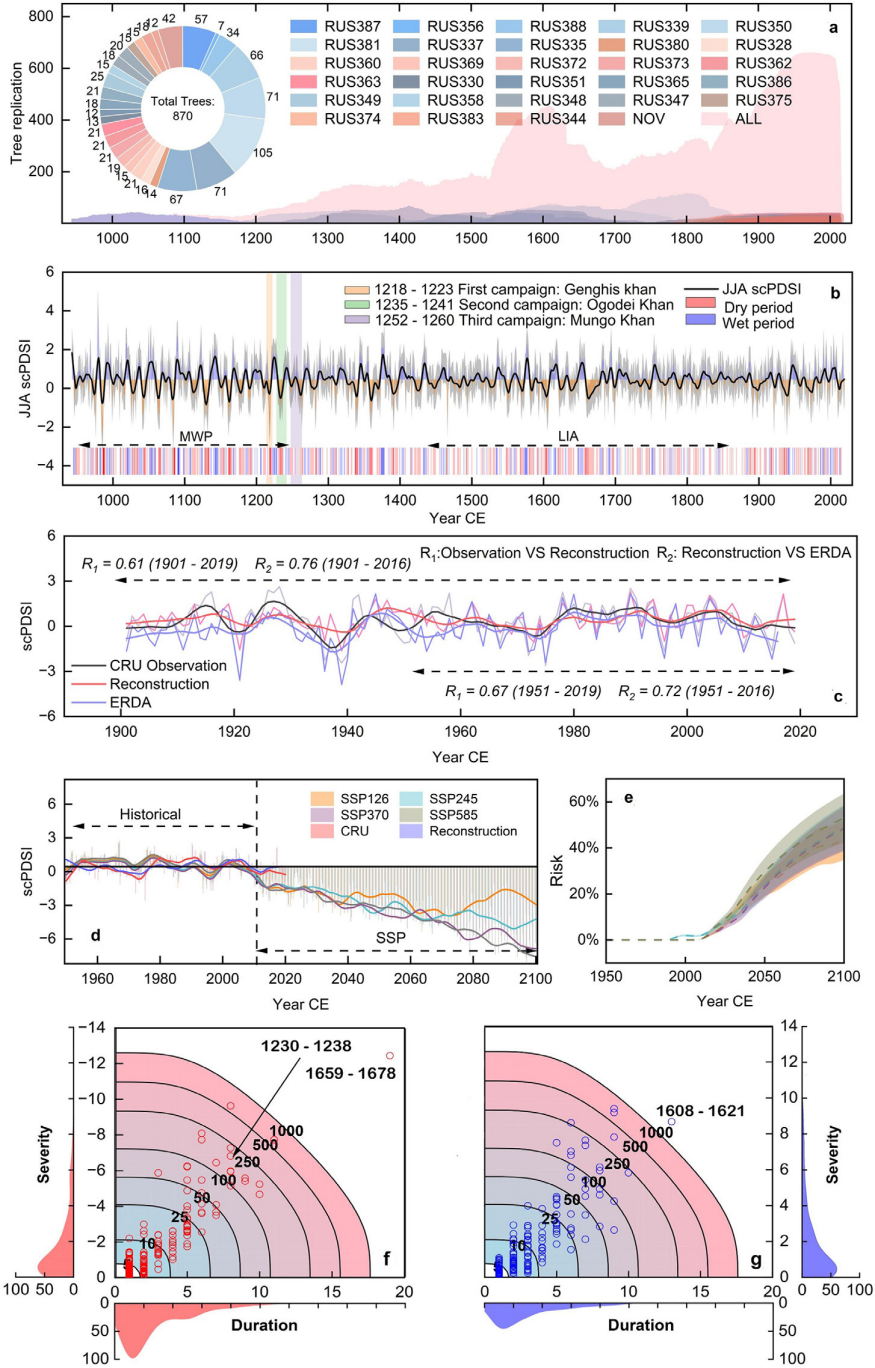
**Reconstruction and prediction of the scPDSI for the Eastern European Plain (EEP).** (a) Depth and distribution of sample size required for the composite chronology. (b) Summer (JJA) scPDSI reconstruction for the EEP since 943, with the application of 10-year filter to highlight decadal trends. The gray shading indicates the error range of ±1 standard deviation (SD = 0.77). The blue/orange shading of the filtered curve is based on the mean of the reconstruction period and it used to visualize changes in dryness and humidity (mean = 0.44). The scPDSI of each year is presented as a heat map to show dry/wet fluctuations. (c) Comparison between the reconstruction, observation, and grid point dataset of scPDSI data during the verification period (Pearson correlation analysis). (d) Predicted future scPDSI changes in the EEP, which include scPDSI changes under four shared economic and societal paths, based on CMIP6 downscaling data. All low-frequency fluctuations are realized using a 10-year low-pass filter. (e) The risk of extreme drought events based on scPDSI changes in the EEP under different SSP scenarios, using the method of Heeter et al. [77], there were smoothed using 15-year moving window low-pass filtering and includes an error interval of 20%–80%. (f) The cumulative severity and duration of droughts (red circles) and (g) wet periods (blue circles) are respectively based on continuous runs of reconstructed scPDSI > 0 and scPDSI < 0 after removing the climatic mean (subtracting the mean value), with the count of each variable provided at the margins (on both left and right sides and at the bottom). The isoclines of return periods (unit is year) (transition from blue to pink filled bands) are derived from the joint probability of the joint function of the severity and duration of wet and dry events.

The rise and fall of civilizations, accompanied by direct and long-lasting societal impacts such as warfare and unrest, have been widely investigated, particularly with regard to the possible and complex links between climate and societal changes [1]. From the 13th to the 14th century, an alliance of nomadic tribes in the hinterland of the Central Asia (CA) steppe established the Mongol Empire across much of Eurasia via ongoing military conquests [2,3]. The largest ever land empire promoted exchange of goods, technology, commodities, and ideology at an unprecedented spatial scale, and even left genetic traces on the people of Eurasia [[4], [5], [6]]. Previous studies not only explored the relationship between increased grassland productivity under a humid climate and the rise of the Mongol Empire [5], but also highlighted the role of intensified streamflow in maintaining a network of oases that enabled the Mongol cavalry to expand westward [2,7]. Although climatic aspects of the Mongol withdrawal from Hungary in 1241/42 CE have been discussed [8], we still lack an understanding of the role of environmental factors that facilitated the Mongols in becoming the largest known steppe empire. Here, we present a millennium-long, tree ring-based reconstruction of water balance changes across the Eastern Europe Plain (EEP) (Figs. 1 and S1), which sheds light on the hydroclimatic background of the Mongol’s westward expansion (Fig. 2). To assess the impact of anthropogenic forcing on current and projected changes in EEP water balance, we use reconstruction results as a basis to validate outputs from various shared socioeconomic pathway scenarios (SSPs) [9]. Through comparisons with ensembles of the Community Earth System Model (CESM), we further identify key mechanisms controlling EEP water balance changes [10].

## 2 Materials and methods

### 2.1 Tree ring data and drought reconstruction

Tree-ring data from European Russia, obtained from the International Tree-Ring Data Bank (ITRDB) and including recently updated series for the region, were used for this study [11] (Figs. 2 and S2; Tables. S1, S2). Sequences from living trees were combined with archaeological samples to construct a composite chronology [12] (Figs. S3, S4). To preserve low-frequency information while mitigating age-related growth trends unrelated to climate, several detrending procedures were tested, including conservative individual detrending techniques, signal-free standardization, and no detrending (raw series) to evaluate their impact on the final chronology. Ultimately, we found that using flexible and variable variance detrending techniques in the RCSigfree program effectively removed age effects from all measurement sequences [13]. First, Friedman variable span smoothing (alpha = 7) was applied to remove the growth trend [14], then double-weighted robust means were calculated to combine all sequences into a single chronology, and finally variance stabilization was applied to mitigate the effects of changing replication and covariance [15]. To ensure a reliable representation of regional, climate-driven variability, a high threshold of expressed population signal (EPS ≥ 0.90) was applied across the full chronology period from 943 to 2019 CE (Fig. S4). Due to the large spatial extent of the composite chronology, we employed gridded meteorological data covering the region, including temperature, precipitation, and the self-calibrating Palmer Drought Severity Index (scPDSI), with the selected grid spanning the interval from 1901 to 2019 CE [16]. Finally, summer (June to August, JJA) scPDSI over the EEP was reconstructed using a simple linear regression model, and model skill was evaluated using standard statistical metrics [17] (Fig. S5).

### 2.2 Characteristics of the reconstruction

**Fig. 3:**
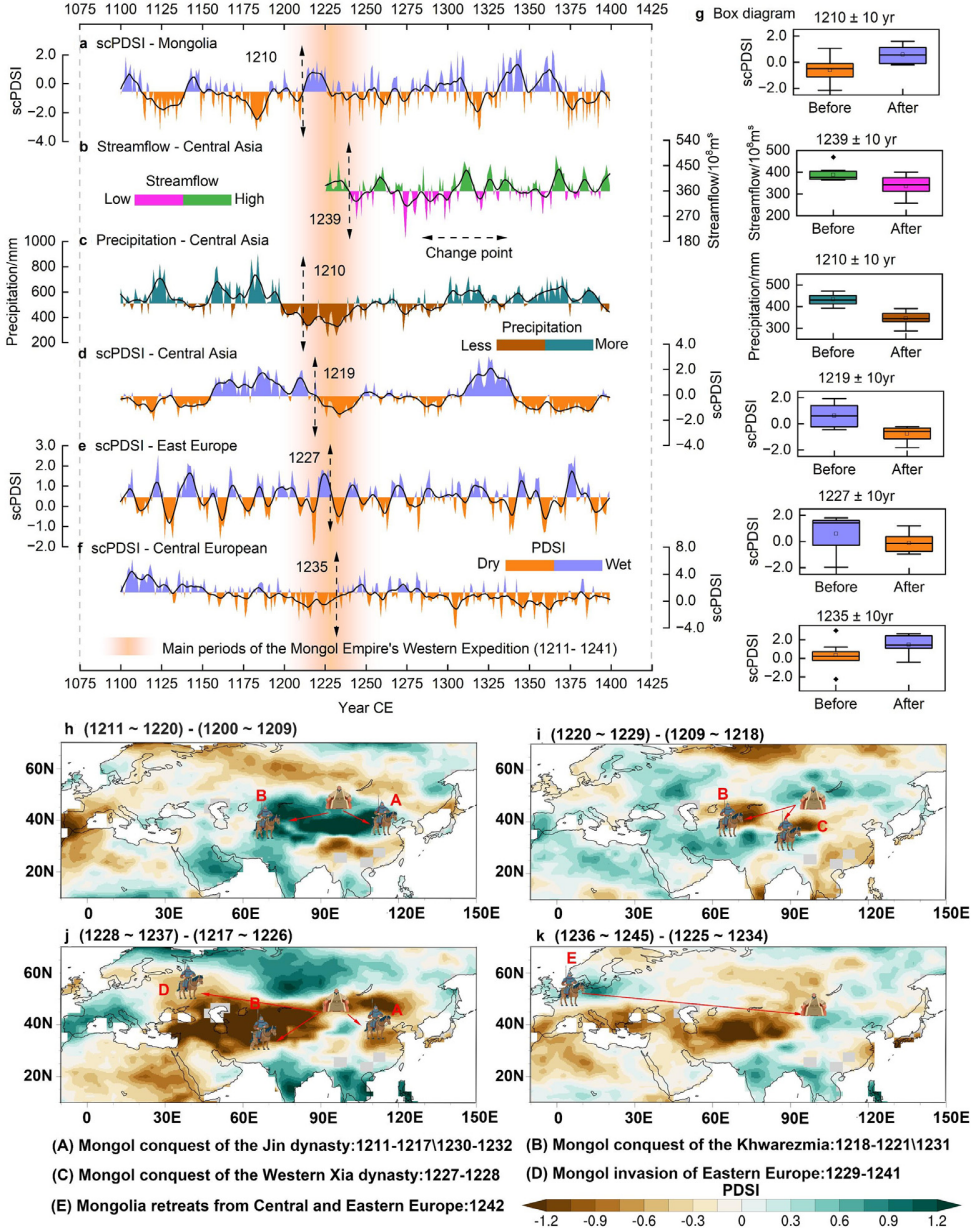
**Climatic and hydrological background of the westward expansion of the Mongolian Empire.** (a) Changes in the warm-season water balance of Mongolia reconstructed by Pederson et al. [5]. (b) Changes in the runoff of Central Asia reconstructed by Chen et al. [2]. (c) Precipitation variability in western Central Asia (Pamir-Alai) for the past 1100 years [47]. (d) Precipitation variability over the past nine centuries in Central Asia (Altai Mountains) [48]. (e) Changes in the water balance in the EEP (this study). (f) Central European water balance reconstruction from tree-ring stable carbon and oxygen isotopes (δ_13_C and δ_18_O) [33]. The above water balance and runoff results reconstructed from tree ring data are only shown for 1100–1399 CE. Change points are used to define dry/wet or high/low runoff shifts. (g) Comparison of boxplots of dry/wet or high/low runoff for 10 years before and after the detected change point, corresponding to the years 1210, 1239, 1210, 1219, 1227, and 1235. (h), (i), (j), (k) respectively show the spatial distribution of PDSI in the first 10 years minus the last 10 years of the main change points (the years 1210, 1219, 1227, 1235). Data are from the Paleo Hydrodynamics Data Assimilation product (PHYDA) developed by Steiger et al. [75], and the approximate spatial locations (red capital letters) and time context of the five main conquered objects on the Mongol Empire’s Western Expedition route are also provided in the figure.

**Fig. 4:**
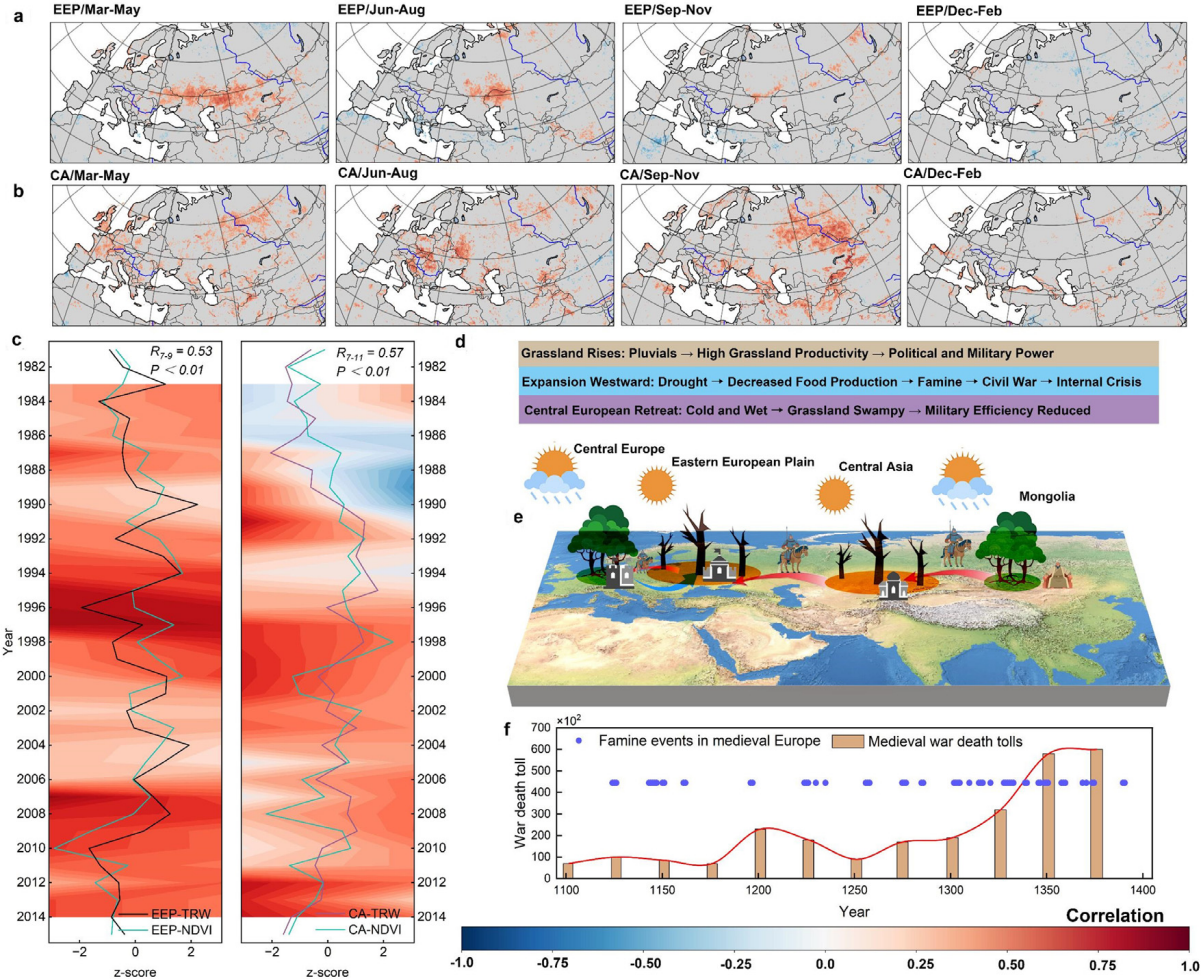
**Interpreting the route selection of the Mongol Empire’s western expeditions from the perspective of changes in water availability and vegetation.** (a) The spatial relationship between TRW of the EEP and NDVI during the instrumental measurement period (1981–2015) was analyzed in terms of four seasons: spring to winter, from left to right. All regions that did not pass the 0.05 significance test were masked. (b) The spatial relationship between TRW and NDVI in the Pamir-Alay region of Central Asia (CA) [47] during the study period was analyzed using the same procedures and calculation methods as described in (a). (c) Comparison between the highest correlation month (July to September) of the TRW and NDVI seasonal combinations. The green line represents the cumulative sum of NDVI from July to September, while the black line represents the TRW. The background shows the interpolated results of the 7-year sliding correlation with original and first-order difference bases. (d) Comparison between the highest correlation month (July to November) of the TRW and NDVI seasonal combinations in the Pamir-Alay region of CA. The green line represents the cumulative sum of NDVI from July to November in the CA, while the black line represents the TRW of the CA. The analysis and calculation procedures are consistent with those described in (c). (e) Conceptual model diagram of the climate background of the rise of the Mongol Empire, the Western Expedition, and the retreat from Central Europe. (f) The occurrence years of famine events in medieval Europe and statistics on war casualties, with war casualties aggregated every 25 years and trend changes presented smoothly using spline functions. The statistical data are sourced from literature published and by Alfani [78] and Sorokin [79].

Using the locally weighted regression method, we determined the decadal-scale fluctuations in the reconstructed sequence, which provided the basis for assessing the occurrence of drought and wet periods, specifically, judging whether a 10-year period was below/above the low-pass filtered average since 943 [18] (Figs. 2 and S7). The mean and standard deviation of the reconstruction period were used to filter years as dry or wet, with the threshold set at ±1σ (σ being the standard deviation) for wet/dry years and ±2σ for extremely wet/extremely dry years [18] (Fig. S7). The climatic state (subtracting the mean) was removed from the reconstruction results and used scPDSI values below or above zero as thresholds for identifying drought and wet events through run theory, thereby calculating their duration and cumulative extent [19]. We estimated the joint probability of the duration and severity of reconstructed droughts and wet events using Copula functions to derive estimates of return periods [19] (Fig. 2). All computational processes and methods can be referenced from the copula package in the R programming language [20]. Potential change points in the time series were first identified based on shifts in the mean and variance of the reconstruction, and then confirmed using the binary segmentation method [21]. Finally, only one change point was retained that encompasses a specific historical period (Fig. 3). Spatial correlation patterns were evaluated using Pearson’s correlation coefficient, and statistical significance was tested using the *t*-test (two-tailed) [17] (Figs. 1, 4 and S6). To identify any periodicities, both the multi-taper method (MTM) and wavelet power spectral analysis were applied, with red noise testing used to assess significance [22] (Fig. S8). Additionally, ensemble empirical mode decomposition (EEMD) was used to identify the intrinsic mode functions of the reconstructed sequence and their inherent characteristics in terms of signal representation on different timescales and frequencies [23] (Fig. S8).

### 2.3 Future projections and multi-factor attribution

**Fig. 5:**
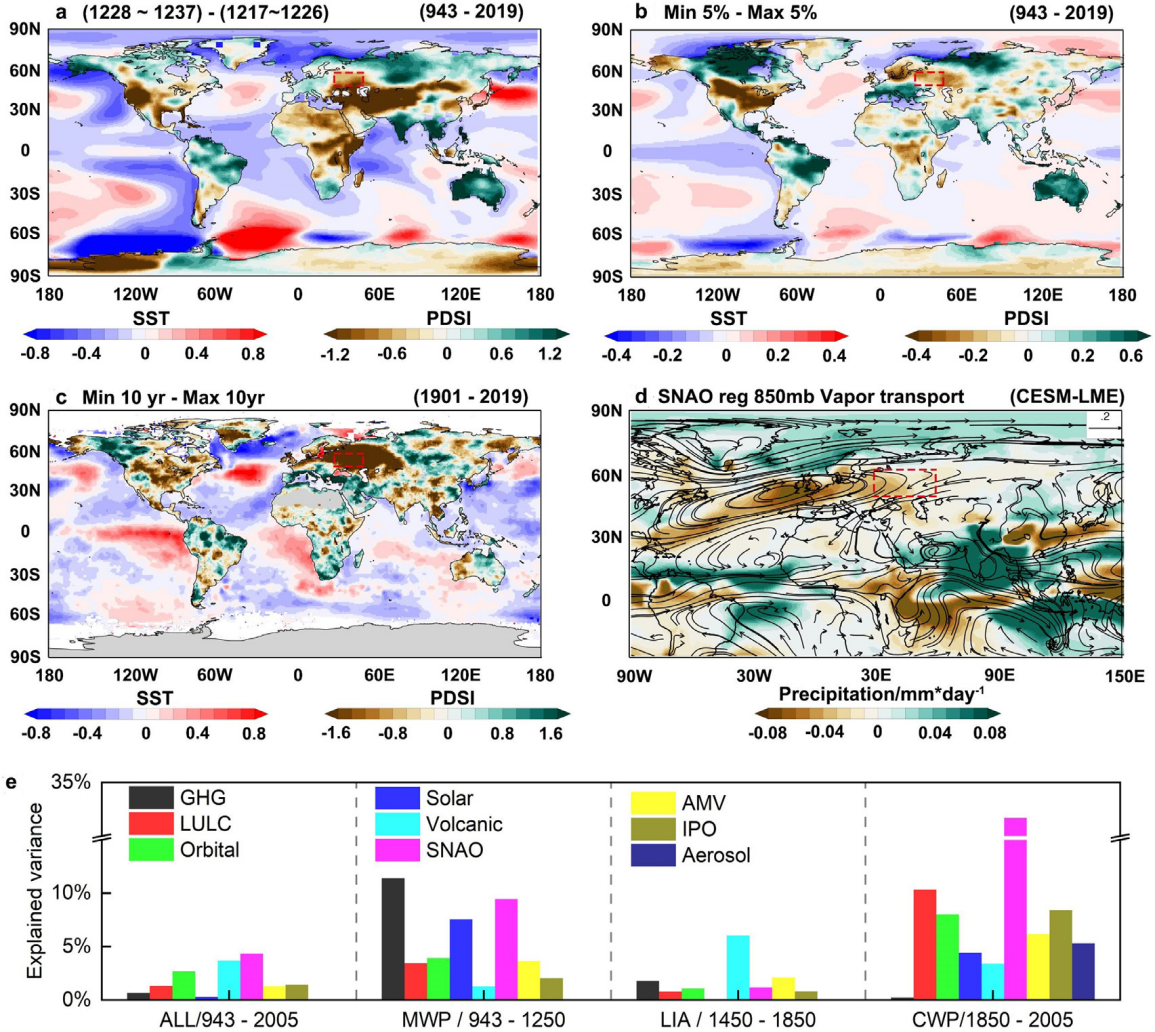
**Maps of sea surface temperature, land-water balance patterns, and multi-factor attribution results.** (a) PDSI for the last decade of 1227 subtracted from the PDSI for the first decade, sourced from the PHYDA [75]. (b) The highest 5% minus the lowest 5% of PDSI from 943 to 2019, sourced from the PHYDA. (c) The highest 10-year scPDSI minus the lowest 10-year scPDSI from 1901 to the mid-year of 2019, using CRU scPDSI and HadISST1 1° reconstruction data sources. (d) Standardized SNAO regressed onto the moisture and precipitation fields, using data from CESM-LME model simulations. The variance explained by different internal and external forcing factors for scPDSI changes calculated from CESM-LME model simulation data across different time periods: the entire interval, the MWP, the LIA, and the CWP. (e) Cumulative variance contribution rate of different internal and external forcing factors of scPDSI changes in the EEP calculated from CESM-LME model simulation data.

To provide a coherent perspective on long-term changes in water balance, we integrated the tree-ring reconstructions with scPDSI from the Coupled Model Intercomparison Project Phase Six (CMIP6) ensemble simulations [9] (Figs. 2 and S12). Temperature and precipitation data from seven CMIP6 climate models were extracted based on a grid distribution of sample points (Table S4). The selected model data were then averaged under four different SSPs for both temperature and precipitation [24]. Prior to computing the scPDSI under the four SSPs, the potential evapotranspiration (PET) was calculated using the Thornthwaite function, which is one of the most widely used formulas in drought index calculations [25,26]. To visualize and evaluate the magnitude of changes between the reconstruction and CMIP6 predicted scPDSI, we used Z-scores to scale the predicted scPDSI from the climate models to fit within the constraints of the mean and standard deviation of the reconstructed scPDSI, following the procedures outlined in Rao et al. [27] (Fig. 2). Additionally, we used multi-member ensemble simulations for the past millennium to quantitatively estimate the contributions of internal climate system variability and external radiative forcing (CESM-LME version 1.1) [10] (Figs. 5 and S13). The external forcings considered in the model experiments encompassed solar variability (Solar), volcanic eruptions (Volcanic), land-use changes (LULC), greenhouse gas concentrations (GHG), and orbital variations (Orbital). The Summer North Atlantic Oscillation (SNAO) [28], Atlantic Multidecadal Variability (AMV) [29], and Interdecadal Pacific Oscillation (IPO) [30] indices were calculated from the model outputs based on standard definitions, representing interannual to decadal variations in sea-air conditions in different ocean regions (Fig. S13). Further information about the CESM-LME modeling project can be found in Otto-Bliesner et al. [10]. The variations in scPDSI over the EEP during the last millennium were attributed using multiple linear regression (MLR) analysis [31]. MLR was used to fit the fully forced scPDSI time series to these explanatory variables [32]. All regressors were smoothed using a 20-year low-pass filter to focus on low-frequency signals.

## 3 Results and discussion

### 3.1 Drought reconstruction of the Eastern European Plain

We conducted a preliminary screening of the tree-ring dataset from the European part of Russia [11], and then selected hydrologically sensitive chronologies from living trees and spliced them to the historical chronology of archaeological and architectural wood to produce a composite chronology [12] (Figs. 2 and S4; Tables. S1, S2). Cross-dating was conducted on this compound chronology, which ensured that it was complete and contained a coherent climate signal [33,34] (Figs. S2, S3). Before generating the composite chronology, we evaluated the reliability of connecting living tree chronologies with archaeological chronologies using a correlation analysis matrix (Fig. S2). The tree-ring width measurements within the common test range passed the 0.05 confidence level test, indicating that the composite chronology can provide a perspective on climate changes in Eastern Europe over the past millennium. Although we successfully established the EEP composite chronology, we still reiterate that there are uncertainties in the process of integrating the chronology of living trees and archaeological trees based on the tree ring width indicator (TRW) [35]. The composite chronology (Fig. S4) is significantly negatively correlated with temperature in the growing season (April to August), positively correlated with precipitation, and significantly positively correlated with the spring and summer scPDSI of the current growing year (Fig. S5). A 60-year moving window correlation analysis was used to test the relationship between the composite chronology and the JJA scPDSI, which was significantly positive, stable, and synchronous on the centennial scale (Figs. 2 and S5, S6). This demonstrates a close long-term relationship between the composite chronology and moisture conditions. The classical mechanism of summer moisture balance changes and tree growth offers a general law explanation, precipitation during the current growing season directly increases soil moisture availability, which compensates for soil moisture loss due to evapotranspiration [36]. However, high temperatures during the current growing season tend to increase both evapotranspiration and the relative humidity, which reduces soil moisture availability [37]. Here we focus on JJA scPDSI, extending the reconstruction back to 943, based on an expressed population signal threshold of 0.90 (Figs. 2 and S4, S5). The developed models demonstrate a good statistical performance, and they collectively explain 38% of the variance during the validation period (Figs. 2 and S5). Spatially, a significant positive correlation pattern between the TRW index and scPDSI extends across the entire EEP (Figs. 1 and S6). Our reconstruction encompasses critical climatic periods during the last millennium on the EEP, including the Medieval Warm Period (MWP, 943–1250 CE), Little Ice Age (LIA, 1450–1850 CE), and Current Warm Period (CWP, 1850–present CE) (Fig. 2). Based on the mean statistical results, the EEP remained in a humid climatic state throughout the interval of study (943–2019 CE; mean scPDSI = 0.44), which is broadly consistent with other reconstructed long-term climate state changes in Eastern Europe and Northern Russia [38] (Fig. S9).

### 3.2 Hydroclimate variation and the westward expansion of the Mongolian Empire

Our reconstruction indicates that the 9-year period from 1230 to 1238 CE witnessed one of the driest periods in the past 1077 years in the EEP, with a cumulative intensity of −6.81 during the 9-year drought beginning in 1230, approaching the 250-year return time estimated based on the joint distribution of drought duration and intensity (Figs. 2 and S7). The millennium scPDSI reconstruction suggests that the EEP has maintained a characteristically moist state. Therefore, it can be anticipated that a severe drought lasting 9 years would cause significant societal disruption (Figs. 2 and S7). Most attempts to link climate change with specific historical events have faced criticism for adopting a simplistic reductionist viewpoint, as climate itself does not cause historical events, but rather, historical events arise from a complex interplay between environmental and human factors; the decisions that humans make can be mediated by and occur in response to environmental conditions [39]. Humans, especially those who have lived in consistently moist climates, have developed a range of adaptation strategies over time, including farming methods and water resource utilization systems [39,40]. Short-term drought stress is tolerable for settled agriculture in moist climate regions, and can be mitigated by rapid adjustments in planting structures to minimize losses [3]. This adaptive resilience ensures the stability of agricultural order without disruption [39]. However, prolonged arid climatic conditions pose a severe challenge to existing adaptation and recovery strategies [33,39,40]. The limited storage capacity fails to address long-term food shortages, and the cumulative agricultural crises, year by year, affect a larger proportion of the population, eventually leading to more significant impacts, even including potential collapse [3]. As documented in the literature, adverse climates and environments causing a decline in grain production in Eastern Europe have triggered famines and wars, depleting the population, horses, and other livestock in principalities like Kyivan Rus [[41], [42], [43]] (Fig. S10). This social unrest provided the opportunity for the Mongols to rapidly invade Eastern Europe [44,45]. Subsequently, from 1237 to 1241 CE, Badu Khan conducted a comprehensive invasion of Ross, and the Duchy of Ross became a vassal of the Mongolian Golden Horde [46].

We have also traced the path of the westward advance of the Mongol Empire, from Genghis Khan to his successor Ogedai Khan, noting the timeline of conflicts and the fall of conquered dynasties (Fig. 1). For comparison, we selected hydroclimate balance reconstruction outcomes for Mongolia [5], Central Asia [2,47,48], and Europe [33]. Using change point analysis, we pinpointed the years corresponding to shifts in the water balance and conducted a decadal-scale comparison of climatic averages [18] (Fig. 3; Table. S3). The rise of the Mongol Empire (1211) and its ultimate retreat from Europe (1242) were accompanied by periods of abundant rainfall, while each expansion involving warfare occurred within the context of drought [2,5,33,47,48]. The change point (based on reconstruction sequence) of 1210 on the Mongolian steppe was followed by 10 years of a consistent warm and humid climate with above average humidity, and the conditions of exceptional humidity contributed to the increased biological productivity of the steppe, which is believed to have contributed to Genghis Khan’s military success and was also an important condition for the formation and rise of political groups [5,19,49] (Fig. 3a). The precipitation reconstruction for CA captures a long-term decrease in precipitation after 1210 (Fig. 3c), and the drought reconstruction also shows a change point in 1219 (Fig. 3d). In 1218, Genghis Khan dispatched an army to cross the Altai Mountains and Tarbagatai Mountains, which ultimately captured Almaryk and successfully conquered the Karachidan dynasty (the Western Liao Empire) [[50], [51], [52], [53]]. Additionally, the Mongol invasion of Khorezm (1219–1221 CE) involved a fierce battle of the imperial army in its attempt to conquer the Islamic world, and it marked the final removal by the Mongols of the major obstacles along the pathway of their western advance [51,[54], [55], [56]]. Subsequently, Genghis Khan assembled troops in Persia and Armenia (1221–1223 CE), divided the Mongolian army into two parts, and crossed Afghanistan to enter northern India and then Russia through the Caucasus, confirming his strategic intentions [[57], [58], [59]].

The transition from nomadic pastoral societies to centralized political entities in the Mongolian plateau (MP) was undoubtedly facilitated by the warm and humid climatic conditions, providing a solid material foundation for its rise [5]. However, the triggering factors behind the Mongol Empire’s westward expansion and rapid conquest of the Eurasian continent cannot be solely attributed to such assumptions [7]. Our analysis reveals that the relationship between water availability and vegetation productivity (Normalized Difference Vegetation Index, NDVI) is closely linked, whether in the EEP or the CA (*R*_7–9_ = 0.53, *R*_7–11_ = 0.57). Significantly positive spatial relationships are observed along the main routes of the Mongol Empire’s western expansion, predominantly covering grassland areas of the Eurasian continent (Fig. 4a,b). Furthermore, we observe dynamic changes in the dry-wet patterns across the Eurasian continent during the Mongol Empire’s westward expansion period (Figs. 3 and S11). Specifically, during the same period (1218–1260 CE), continuous arid climatic conditions were evident in the EEP, CA, and MP. These findings, viewed from the perspective of spatial dry-wet patterns and grassland vegetation productivity, lead us to conclude that drought played a significant role in driving the Mongol Empire’s westward expansion and rapid conquest of the Eurasian continent. Drought conditions led to dry grassland environments, reduced soil moisture, decreased vegetation productivity, and the flat grassland was like a hardened highway, facilitating the swift maneuverability of Mongol cavalry [8,60]. However, such drought conditions also weakened the less mobile local agricultural societies [61], compounded by internal turmoil in the conquered regions [40] (Fig. 4f), ultimately enabling the Mongol Empire to swiftly conquer the Eurasian continent within a short period. Although caution must be exercised in inferring the nature of the complex mechanisms of the spatial and temporal interactions between humans and the environment [59,62,63], the relationship between drought and the Western Expedition of the Mongol Empire remains a notable case study, in which tree ring data are used to validate historical records and explore the possible role of relatively minor environmental factors in major sociocultural, political and economic phenomena [64].

### 3.3 Drought in the Eastern European Plain: examine the past and predict the future

Although empirical data and model simulations both indicate that Europe’s climate is becoming increasingly arid, unfortunately, current research has not delved deeply into the attribution of drought changes in the EEP, nor has it placed recent events within the context of past natural variations and future changes expected due to anthropogenic climate change [33,[65], [66], [67]]. Mean calculations and Kernel density distribution analysis reveal that over the past 1000 years, the EEP was generally humid but with a long-term trend towards arid conditions (Figs. 2 and S8). The intensity distributions of dry and wet periods are largely consistent, but in terms of duration, droughts are mainly concentrated in short periods of less than 2 years, while wet periods are more evenly distributed [68] (Fig. 2f,g). The expected recurrence times calculated based on the joint probability of intensity and duration show that most drought or wet periods complete their return time within 100 years, but wet periods exceeding 100 years occur more frequently than droughts [19]. Notably, no wet or dry period representatives of the CWP have been recorded within the recurrence time thresholds of 500 or 1000 years. Cumulative events and their ratios to the duration period revealed that the probabilities of extreme dry/wet events during the MWP (39.6%) and CWP (22.9%) were higher than during the LIA (21.2%), it may be the result of the warmer climate effect under the warm period background [33] (Fig. S7d).

We found that climate and radiation factors influencing future drought were contingent on both timescales and uncertainties. Under the four forcing scenarios, by 2100, droughts will continue to occur on interannual to interdecadal scales, both in terms of trend and frequency, which suggests the EEP will face continued drought risks in the 21st century (Figs. 2e and S12). The underlying multidecadal-to centennial-scale drought trend is determined by the timing and magnitude of long-term changes in temperature and precipitation. However, it is apparent that the simulated increases in precipitation, in some scenarios, do not compensate for the continued rise in summer surface air temperatures through 2100 (Fig. S12). The rising temperatures, together with the associated increase in evaporative demand, drive the system towards persistent aridity within all forcing contexts. But there are some reversals, for example, against the background of weak radiative forcing (SSP1–2.6, with radiative forcing reaching 2.6 W/m^2^ in 2100), the EEP will reverse trend to progressive moisture compensation by 2070 (Figs. 2 and S12), but it still more arid than the historical overlap period, which shows that temporal variability increases the uncertainty of EEP drought predictions [24].

Similar to previous hydrological studies in Europe, our spatial correlation analysis of the EEP reconstruction shows that the drought structures in Eastern and Northern Europe versus those in Southern Europe are the opposite [66,69,70] (Figs. 1, S6). This dipole-like drought structure may be caused by changes in the SNAO, the principal atmospheric circulation that regulates the climate of Europe [71,72]. The EEMD results demonstrate that the Intrinsic Mode Functions (IMFs) contributing to the major variance also show decadal oscillations (9.5–16.5 years) (Fig. S8). The SNAO is regarded as the dominant teleconnection pattern for European summer precipitation and heat waves on interannual and interdecadal scales [71,73]. High-altitude tropospheric westerly jets and storm tracks moving from north to south create anomalies in the European summer moisture balance [74]. Maps of drought periods minus wet periods at different scales show a consistent pattern of drought in the EEP and at the same time a meridional tripolar sea surface temperature anomaly pattern in the North Atlantic [71,75] (Figs. 4 and S6). The return of the SNAO at the 850 hPa water vapor transport field shows a pronounced anticyclone that causes the transport of dry and warm air from northern Africa to southeastern Europe, resulting in extremely dry and hot summers in the EEP [66,67] (Fig. 5). Multiple linear regression attribution analysis of scPDSI simulations from the CESM-LME model shows that the SNAO maintains a relatively high contribution across all time periods, especially during the modern warming period, and it also makes the largest variance contribution among the specified internal and external forcing factors (Figs. 5e and S13). Therefore, our study calls for the enhancement of predictive capabilities for internal variability patterns (SNAO) to improve the forecast of scPDSI on a decadal scale in the EEP. Further understanding and predicting the recent evolution of SNAO and other decadal modes of internal variation will lead to more accurate predictions of drought changes in Europe under the backdrop of global warming.

## 4 Conclusion

This study presents a high-resolution, millennium-long reconstruction of summer water balance across the EEP, revealing persistent hydroclimatic variability and its far-reaching sociohistorical implications. Our results demonstrate that the 1230s marked one of the most severe and prolonged droughts of the past 1077 years in the EEP, coinciding with the Mongol Empire’s westward expansion. The alignment between arid climatic conditions and key historical transitions suggests that drought likely played a critical, albeit indirect role, by weakening sedentary agricultural societies and simultaneously creating favorable conditions for the maneuverability of Mongol cavalry across hardened grasslands. By integrating hydroclimatic reconstructions from CA, MP, and Eastern Europe, we identify a synchronous pattern of drought along the primary routes of Mongol military campaigns during the early 13th century. These findings offer novel evidence that sustained climatic stress may have amplified the geopolitical volatility of the region, enabling rapid military advances and contributing to the transformation of Eurasia’s political landscape. Our analysis also attributes historical drought variability in the EEP to large-scale modes of internal climate variability, particularly the SNAO, which emerges as the dominant driver of decadal-scale hydroclimatic fluctuations. Future projections based on CMIP6 simulations reveal that anthropogenic warming is likely to exacerbate aridity across the EEP, with rising temperatures outweighing any potential compensatory increases in precipitation. Collectively, our findings underscore the complex interplay between climate variability and societal change, and provide an integrative framework that bridges paleoclimatic evidence, model projections, and historical analysis to better understand the multifaceted role of climate in shaping human history and guiding future adaptation strategies.

## Data availability

The tree-ring data can be found in the International Tree-Ring Data Bank (ITRDB) (https://www.ncei.noaa.gov/roducts/ppaleoclimatology/tree-ring). The CRU TS Version 4.04 high-resolution meteorological grid data set can be obtained from the Climatic Research Unit website (https://crudata.uea.ac.uk/cru/data). The Paleo Hydrodynamics Data Assimilation product (PHYDA) can be downloaded from the Zenodo platform (https://zenodo.org/records/1198817). The normalized difference vegetation index (NDVI) used in this study is derived from the Standardized Difference Vegetation Index of the Global Inventory Modeling and Mapping Studies (UMD GIMMS NDVI 3 g analysis) produced by the National Center for Atmospheric Research (https://www.ncei.noaa.gov/metadata/geoportal/rest/metadata/item/gov.noaa.ncdc:C01558/html). All data from CMIP6 simulations used in our analyses are freely available from the Earth System Grid Federation (https://esgf-node.llnl.gov/search/cmip6/). The Community Earth System Model: Last Millennium Ensemble Project data (CESM-LME) can be obtained from the website (https://www.cesm.ucar.edu/community-projects/lme/data-sets). Further analytical data relevant to the manuscript can be obtained from the authors upon request.

## Code availability

The code to perform the current analysis is available from the main corresponding author upon request.

## CRediT authorship contribution statement

**Weipeng Yue:** Writing – original draft, Visualization, Validation, Software, Resources, Project administration, Methodology, Investigation, Funding acquisition, Formal analysis, Data curation, Conceptualization. **Feng Chen:** Writing – review & editing, Supervision, Software, Resources, Project administration, Methodology, Funding acquisition, Formal analysis, Data curation, Conceptualization. **Olga Solomina:** Writing – review & editing, Validation, Resources, Methodology, Formal analysis, Data curation. **Jan Esper:** Writing – review & editing, Supervision, Methodology, Conceptualization. **Nicole K. Davi:** Writing – review & editing, Supervision, Methodology, Data curation. **Ulf Büntgen:** Writing – review & editing, Supervision, Methodology, Formal analysis, Data curation. **Shijie Wang:** Visualization, Methodology, Investigation, Formal analysis, Data curation, Conceptualization. **Vladimir Matskovsky:** Methodology, Investigation, Data curation. **Caroline Leland:** Writing – review & editing, Validation, Resources, Methodology, Funding acquisition. **Leonid Agafonov:** Resources, Data curation. **Max C.A. Torbenson:** Writing – review & editing, Supervision, Methodology, Investigation, Formal analysis. **Magdalena Opała-Owczarek:** Writing – review & editing, Resources, Funding acquisition, Data curation. **Mao Hu:** Visualization, Software, Conceptualization. **Marina Gurskaya:** Resources, Data curation. **Zulfiyor Bakhtiyorov:** Visualization, Methodology. **Xiaoen Zhao:** Visualization, Methodology, Data curation. **Yang Xu:** Visualization, Investigation, Data curation. **Heli Zhang:** Visualization, Investigation, Data curation. **Youping Chen:** Visualization, Data curation. **Fahu Chen:** Writing – review & editing, Supervision, Resources, Conceptualization.

## Declaration of competing interest

The authors declare that they have no conflicts of interest in this work.

## Appendix Supplementary materials

Image, application 1
