# Supplementary material for: Drought facilitated the westward expansion of the Mongol Empire in the 1230s
Source: Fundam Res. 2025 Sep 3;6(4):2417–26. doi: 10.1016/j.fmre.2025.08.010 (PMC13424159; doi:10.1016/j.fmre.2025.08.010)
Supplement: Supplementary file 1 [file mmc1.docx]

**Supplementary Material**

**Drought facilitated the westward expansion of the Mongol Empire in the 1230s**

Weipeng Yue *^a, b^*, Feng Chen *^a, c, d *^*, Olga Solomina *^e, f *^*, Jan Esper *^b, g^*, Nicole K. Davi *^h, i^*, Ulf Büntgen *^g, j, k^*, Shijie Wang *^a^*, Vladimir Matskovsky *^e^*, Caroline Leland *^h, i^*, Leonid Agafonov *^l^*, Max C.A. Torbenson *^b^*, Magdalena Opała-Owczarek *^m^*, Mao Hu *^a, c^*, Marina Gurskaya *^l^*, Zulfiyor Bakhtiyorov *^a, i, n^*, Xiaoen Zhao *^a, c^*, Yang Xu *^a^*, Heli Zhang *^a, d^*, Youping Chen *^a^*, Fahu Chen *^o, p, q *^*

*a Yunnan Key Laboratory of International Rivers and Transboundary Eco-Security, Institute of International Rivers and Eco-Security, Yunnan University, Kunming, 650500, China*

*b Department of Geography, Johannes Gutenberg University, Mainz, 55122, Germany*

*c Southwest United Graduate School, Kunming, 650500, China*

*d Key Laboratory of Tree-ring Physical and Chemical Research of the China Meteorological Administration / Xinjiang Laboratory of Tree-ring Ecology, Institute of Desert Meteorology, China Meteorological Administration, Urumqi, 830002, China*

*e Institute of Geography, Russian Academy of Sciences, Moscow, 119017, Russia*

*f National Research University Higher School of Economics, Moscow, 109028, Russia*

*g Global Change Research Institute, Czech Academy of Sciences, Brno, 60300, Czech Republic*

*h Department of Environmental Science, William Paterson University, Wayne, 07470, USA*

*i Tree-Ring Lab, Lamont-Doherty Earth Observatory, Columbia University, New York, 10964, USA*

*j Department of Geography, University of Cambridge, Cambridge, CB2 3EN, UK*

*k Department of Geography, Faculty of Science, Masaryk University, Brno, 61137, Czech Republic*

*l Laboratory of Dendrochronology, Institute of Plant and Animal Ecology, Russian Academy of Sciences, Yekaterinburg, 620144, Russia*

*m Institute of Earth Sciences, Faculty of Natural Sciences, University of Silesia in Katowice, 41-200, Poland*

*n Khujand Science Center, National Academy of Sciences of the Republic of Tajikistan, Khujand, 735714, Tajikistan*

*o ALPHA, State Key Laboratory of Tibetan Plateau Earth System, Environment and Resources (TPESER), Institute of Tibetan Plateau Research (ITPCAS), Chinese Academy of Sciences (CAS), Beijing, 100101, China*

*p College of Resources and Environment, University of Chinese Academy of Sciences, Beijing, 100049, China*

*q MOE Key Laboratory of Western China's Environmental System, Lanzhou University, Lanzhou, 730000, China*

**E-mail of corresponding author:**

Feng Chen: feng653@163.com

Olga Solomina: olgasolomina@yandex.ru

Fahu Chen: fhchen@itpcas.ac.cn


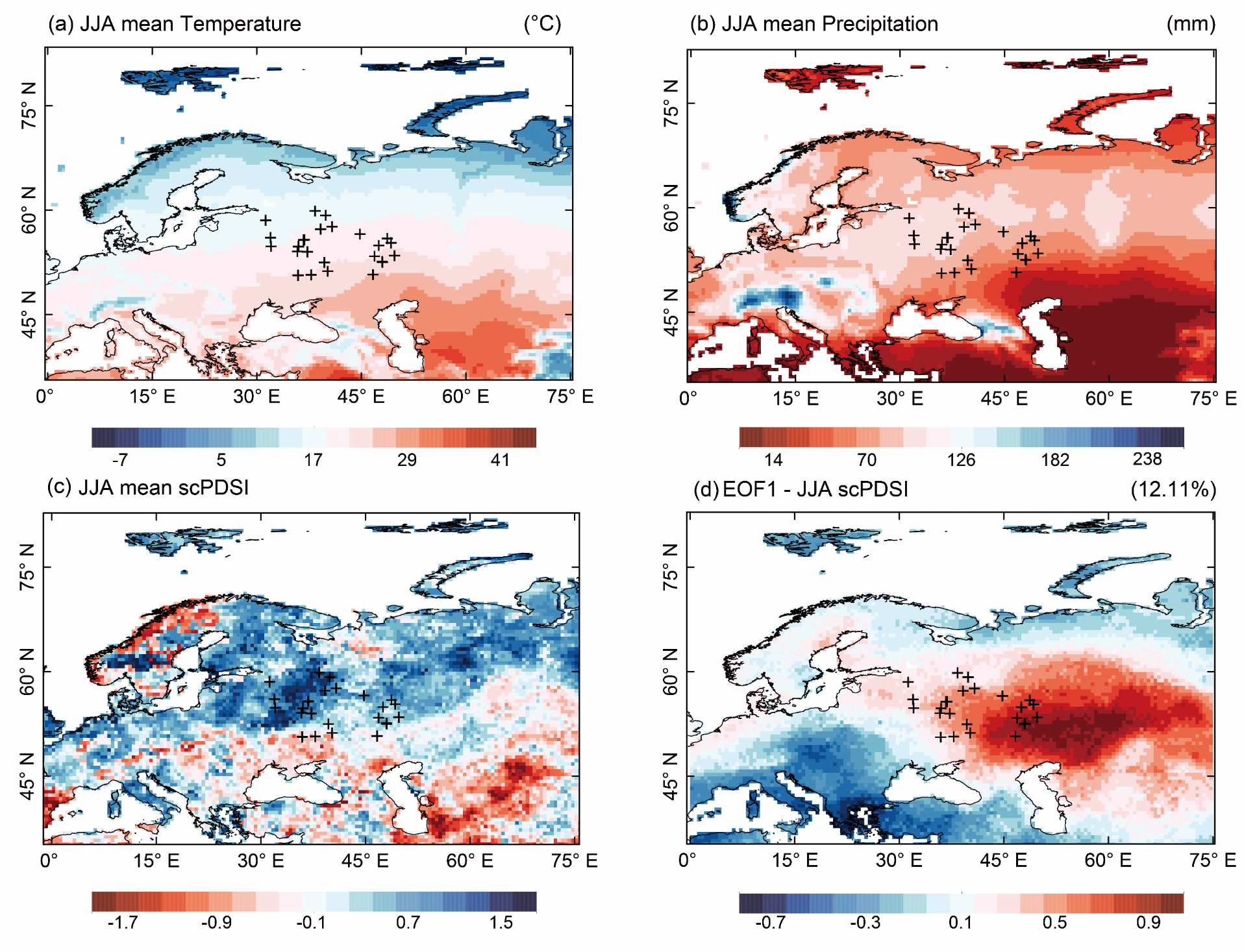


**Supplementary Fig. 1 Overview of summer (June to August) climate in the study area from 1901 to 2019.** (a) Mean temperature distribution in summer. (b) Mean total precipitation distribution in summer. (c) Distribution of wet and dry conditions in summer. (d) Spatial distribution of the first mode (EOF1, Variance explained = 12.11%) of an EOF analysis of scPDSI (Beckers and Rixen, 2003). The spatial range of the EOF analysis is 0°E–75°E and 35°N–85°N. The black crosses are tree ring sampling sites. The mean summer temperature in the study area ranges from 14 °C to 26 °C, and mean summer precipitation ranges from 42 mm to 92 mm. Summers from 1901 to 2019 were generally humid. The variance explained by EOF1 of scPDSI is 12.11%, suggesting that the spatial pattern of wet and dry variability within the sampling area is largely coherent. All the above grid meteorological data are from the CRU (University of East Anglia) (Kottek et al., 2006), with the spatial resolution of 0.5° × 0.5°.


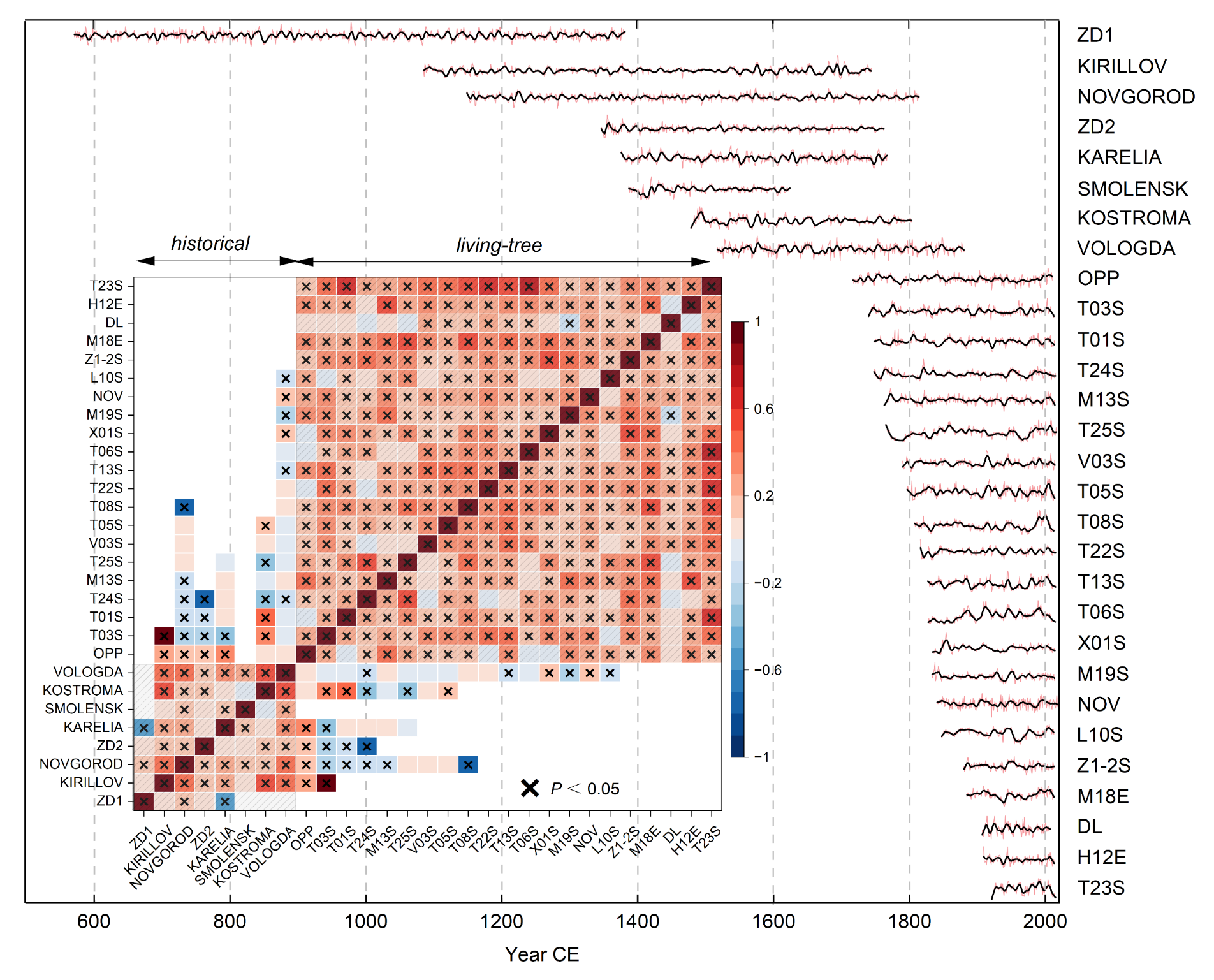


**Supplementary Fig. 2 Results of correlation analysis of all the tree-ring measurement sequences.** Ten-year low-pass filtering was applied to all chronologies to capture interdecadal changes (Cook et al., 2000). In the heat map, the black crosses indicate that the correlation is significant at the 0.05 level. Initially, the measurement sequence of each living tree sample in the database was subjected to a moisture sensitivity test, and then the developed chronologies were detrended using ARSTAN software. Within the instrumental period (1901–2019 CE) the moisture sensitivity of each living tree chronology was assessed and 21 chronologies were retained (Cook, 1986). Given the substantial variations in interval lengths among the archaeological tree-ring series and in sample sizes, we exercised caution in using the archaeological tree-ring measurement sequences (Manning, 2023). Similar to the measurement sequences of living trees, we developed tree-ring index chronologies for each archaeological tree measurement sequence in the database. First, we performed preliminary statistical verification using COFECHA software, excluding chronologies with poor dating quality from the archaeological tree-ring measurement sequences (Grissino-Mayer, 2001). We then assessed whether the historical chronologies could be further used based on their similarity to the living tree chronologies and reference chronologies within the overlapping common intervals.


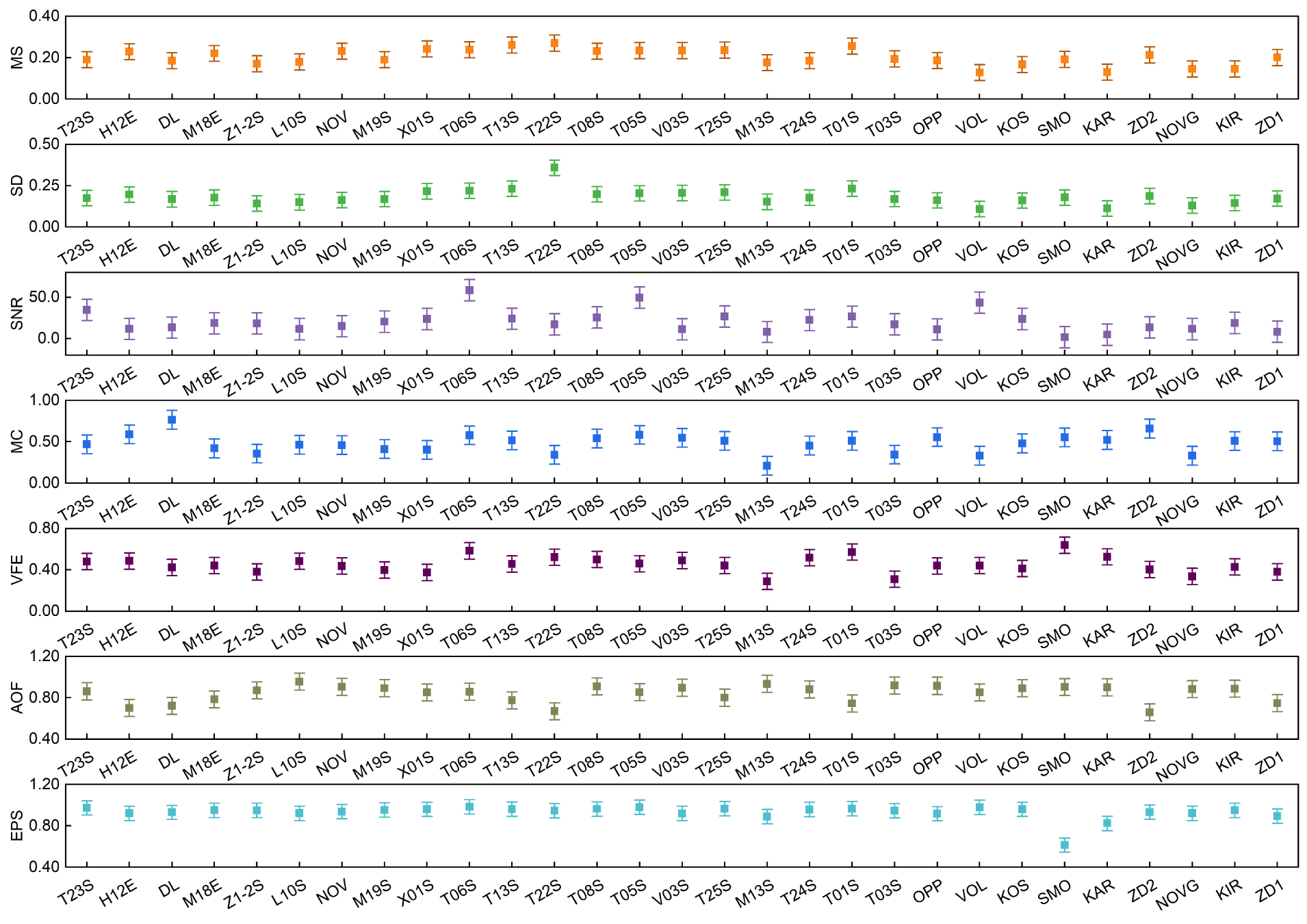


**Supplementary Fig. 3 Results of statistical analysis of all chronologies.** Note: SD is the standard deviation, SNR is the signal-to-noise ratio, MC is the mean correlation with the master series, VEE is the variance of the first eigenvector, AOF is the first-order autocorrelation, and EPS is the expressed population signal (Cook and Kairiukstis, 2013). The statistical results for most of the chronologies show well-distributed intervals, encompassing a large number of climate signals, and display a climatically sensitive response. However, there are a few unsatisfactory statistical results, possibly due to inadequate sample sizes, indicated by an overall decrease in signal strength.


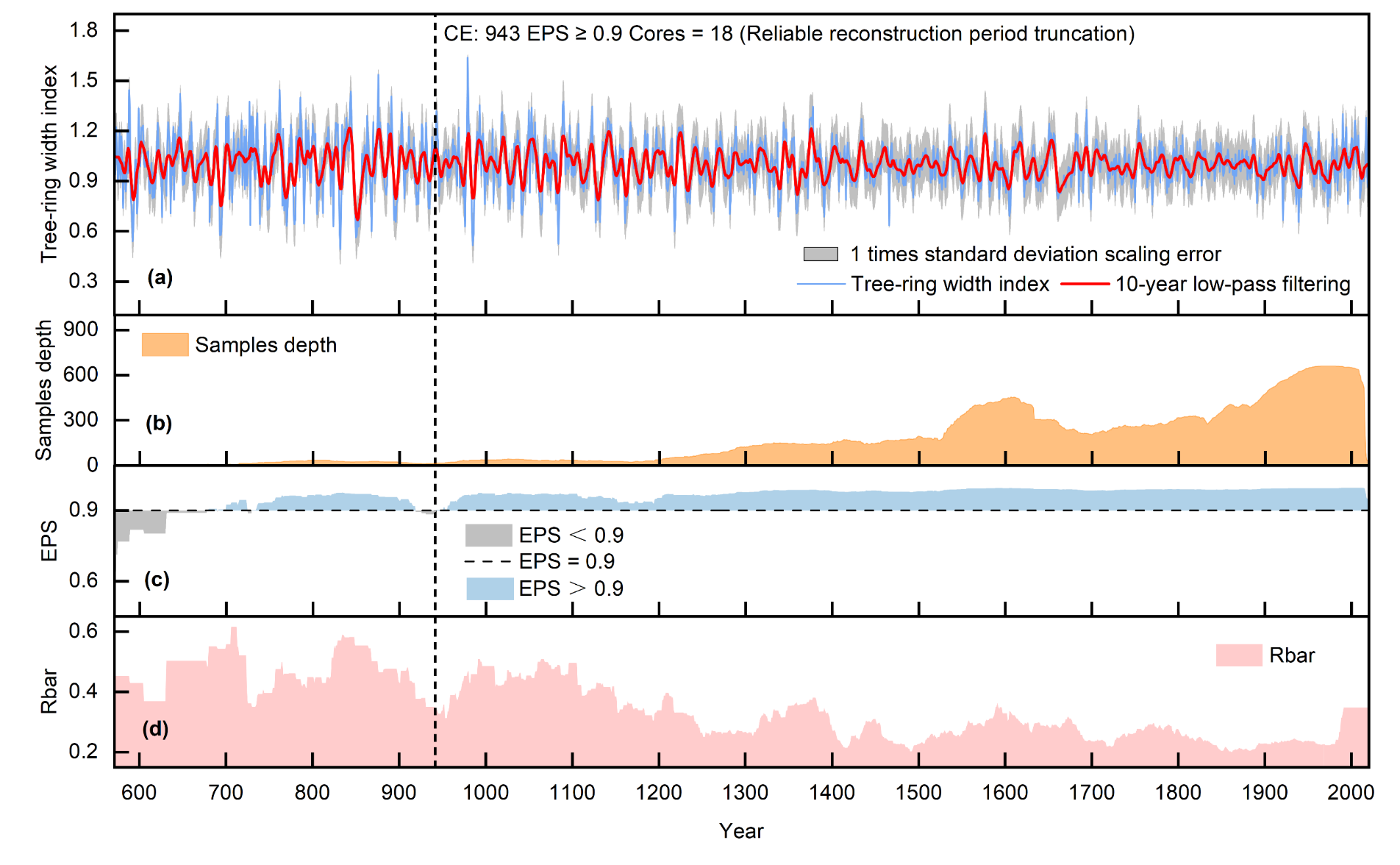


**Supplementary Fig. 4 Composite chronology for the Eastern European Plain (EEP).** (a) Composite chronology for the EEP. The red line represents the results of 10-year low-pass filtering (LOWESS), and the gray shading indicates the annual tree-ring index, with 1σ uncertainty (population standard deviation σ = 0.145). SsfStb denotes the mean variance-adjusted signal-free regional chronology (Yue et al., 2023). (b) Results of the merging of the sample depths of all individual point chronologies and their extension to the present-day (indicated by the orange shading). (c) Expressed Population Signal (EPS) statistic, calculated over 51 years and lagged by 50 years, using a threshold level of 0.90 (blue shading represent regions with values > 0.90, and grey shading represents the rest). (d) Mean Inter-Series Correlation (Rbar) record. Although we have successfully established a composite chronology for the EEP, we must still reiterate the numerous uncertainties involved in the process of merging living tree and archaeological remnant tree chronologies based on TRW proxies (Büntgen et al., 2010). For instance, starting from the initial source, differences between temperate tree species lead to complex physiological interpretations (Friedrichs et al., 2009). Next, there is a lack of precise understanding about the origin of archaeological timber (Haneca et al., 2009). Finally, the standardization methods for tree rings are flexibly applied in preserving low-frequency signals (Esper et al., 2005). These results can lead to the amplification of noise in high-frequency signals, variance inflation in low-frequency signals, and distortion of climate signal recordings. Regarding the physiological confusion effects caused by mixing tree species, here we primarily use common European tree species such as *Oak*, *Pine*, and *Spruce*, which have been widely utilized in dendroclimatology and hydrological reconstruction (Büntgen et al., 2011). The precise understanding of wood sources, constantly changing forest management, and site ecology over time can also introduce uncertainties, though the issue here seems not substantial. The selection based on transportation distance and cost considerations usually limits the source of timber to surrounding major forest areas. Moreover, Eastern Europe-Moscow, as a traditional resource-rich area of northern forests, coupled with rapid forest age renewal and cold climate, provides well-preserved sub-fossil quality. Lastly, our focus in establishing the chronology is on the process. We opt for the RCS method to eliminate the so-called "segment length curse," then use Osborn et al. (1997) method to stabilize variance, and finally apply a population signal threshold of over 0.85 to constrain the consistent range of reliable signals.


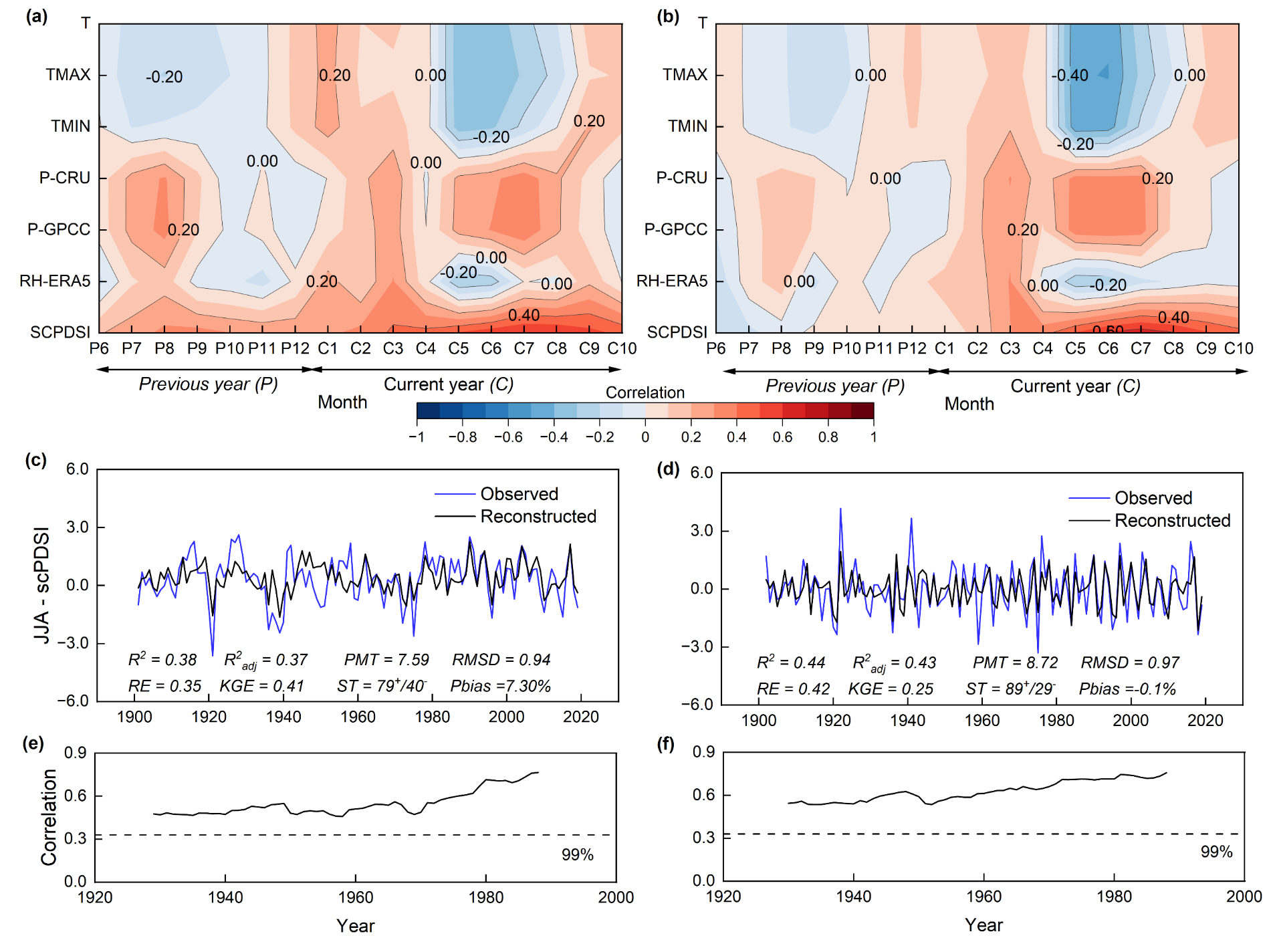


**Supplementary Fig. 5 Relationship between the tree-ring width index and meteorological variables during the observation period, and comparison of the scPDSI reconstruction and observations.** The original data are on the left, and the results after first-order differencing are on the right. Pearson correlation was used to explore the relationship between the tree ring width index and monthly climatic factors during 1901–2019 CE (Biondi and Waikul, 2004). These factors include mean temperature (T), mean minimum temperature (TMIN), mean maximum temperature (TMAX), precipitation (P-CRU and P-GPCC), relative humidity (RH-ERA5), scPDSI, and other related meteorological elements (Hersbach et al., 2020; Schneider et al., 2016). The current year's climate may have a lagged impact on next year's growth and therefore we included climate data from June of the previous year to October of the current year (D'Arrigo et al., 2008). The analysis results are shown in (a) and (b). The TRW of the composite chronology is positively correlated with precipitation in the previous year’s winter, spring, and summer, or the current year; and negatively correlated with the summer temperature or relative humidity of the previous year or current year. Additionally, temperature and precipitation in winter and spring are both positively correlated with the scPDSI for all months or seasons. The highest correlation coefficient is 0.609 in July during the summer, and the correlation coefficient after first-order differencing is 0.667. These results indicate that hydrologically sensitive Scots pine, spruce, or oak in the EEP are affected by summer drought stress during their annual radial growth process. High temperatures during the growing season cause soil water loss, increased plant respiration, and excessive nutrient consumption (Brouder and Volenec, 2008). However, timely precipitation replenishment can mitigate the adverse effects of rising temperatures. The positive correlation between temperature or precipitation in winter and spring and the TRW suggests that limited warming accelerates snow melting and supplements soil water consumption before the growing season (Skomarkova et al., 2006). Comparison of reconstructed and observed scPDSI is shown in (c) and (d). Based on the stable relationship between the tree ring width index and scPDSI from 1901 to 2019 CE, we converted the tree ring width index to the scPDSI index using a linear regression model:

Y_scPDSI_ = 6.067×X_i_ - 5.6189

The model explains 38% of the variance, and the explained adjusted variance is 37%, while after first-order differencing the explained variance increased to 44%. Various statistical methods were used to test the stability and reliability of the model, including RE (reduction of error), ST (sign test), PMT (product means test), RMSD (Root Mean Square Deviation), KGE (Kling-Gupta efficiency), and Pbias (Relative bias in percent) (Chanapathi et al., 2018). Moving window correlation (width = 60 years) was used to examine the dynamic effect of the model signal simulation. The results are shown in (e) and (f). As the step length varied, the relationship between the observation and reconstruction results remained significant and stable; however, it may be influenced by instrumental factors. Due to the absence of test data, the correlation in the early stage is lower than in the later stage, resulting in a lower overall variance explained by the model.


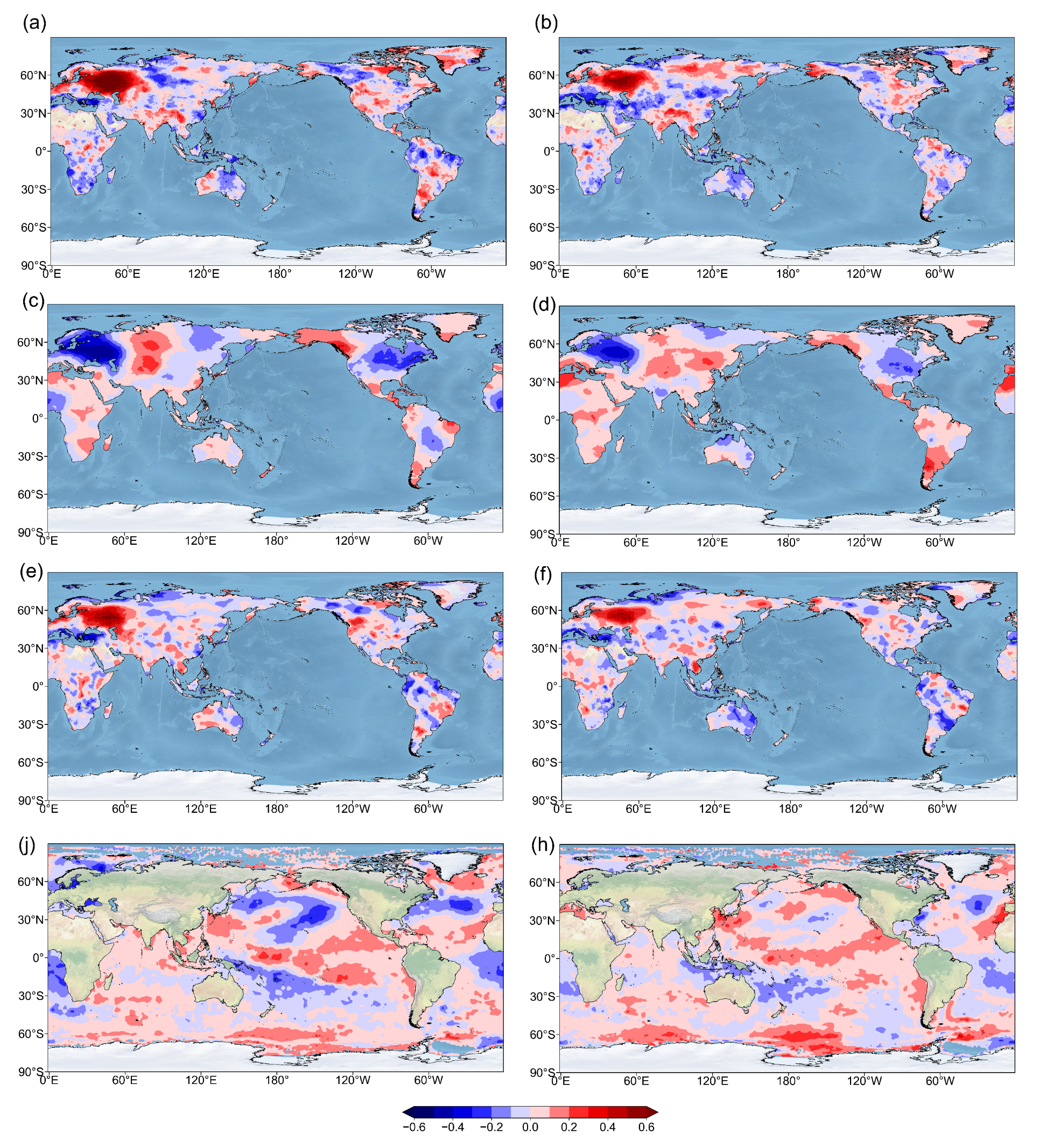


**Supplementary Fig. 6 Results of spatial correlation analysis of the scPDSI and various meteorological and oceanographic indices.** The left side shows observations and the right side the reconstructed data. (a) and (b) illustrate the spatial correlation fields between the observed or reconstructed scPDSI and the global JJA scPDSI grid dataset. (c) and (d) show the spatial correlation fields between the observed or reconstructed scPDSI and the global JJA mean temperature grid dataset. The correlation fields in (e) and (f) show the spatial correlation between the observed or reconstructed scPDSI and the global JJA total precipitation grid dataset. (g) and (h) show the spatial correlation fields between the observed or reconstructed scPDSI and the global JJA sea surface temperature (SST) grid dataset. All spatial correlation analysis results are shown as dots within the 95% confidence area. The results demonstrate that both the observed and reconstructed scPDSI show consistent spatial correlation distributions with various meteorological and oceanographic indices. This validates the stability and reliability of the reconstruction results and highlights the ability of the EEP composite chronology to capture large-scale spatial signals. In particular, the spatial correlation with SST reveals a teleconnection mechanism between the North Atlantic Ocean and the mid- and low-latitude Pacific Ocean regarding wet and dry variations in the EEP (Wang et al., 2015).


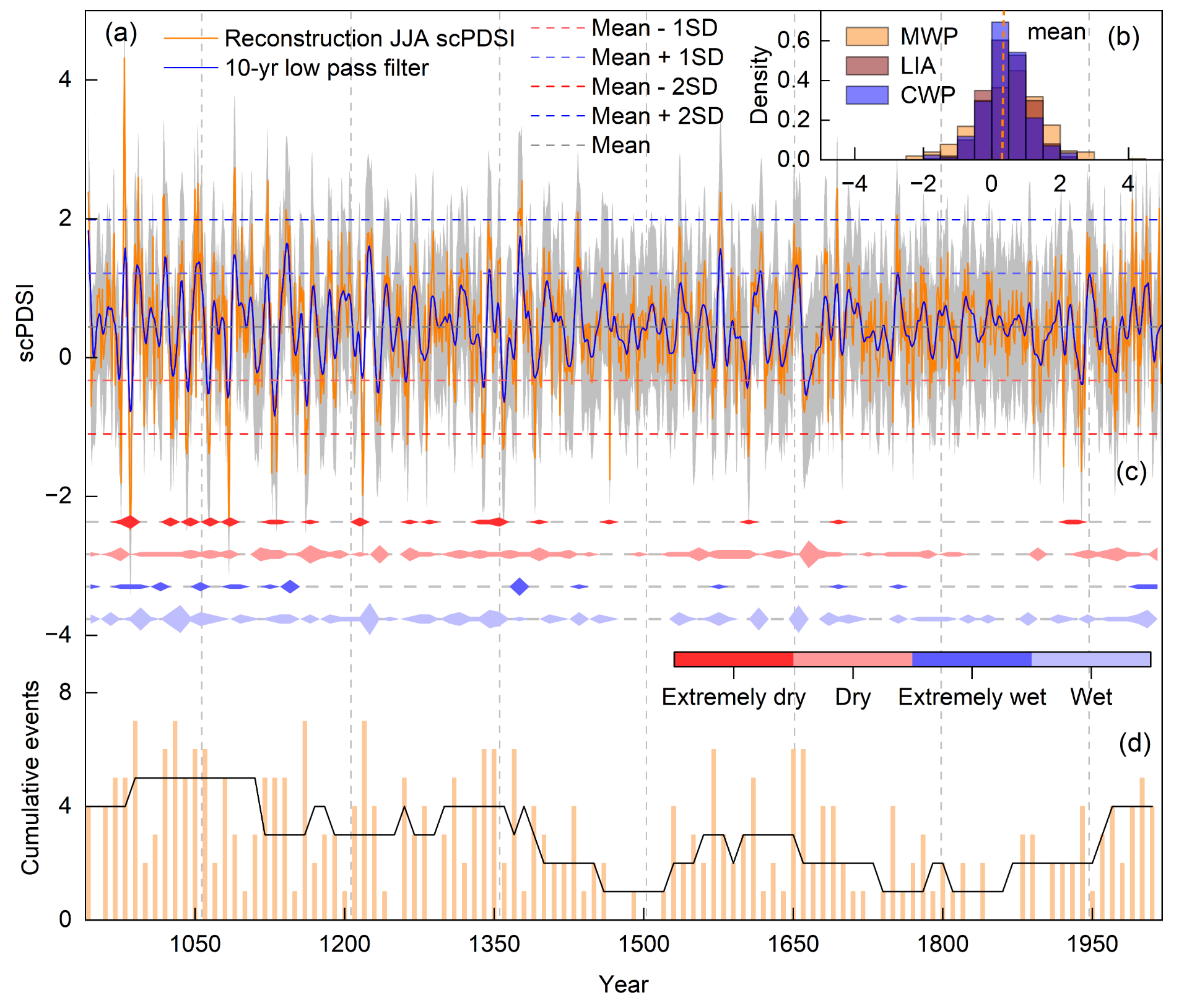


**Supplementary Fig. 7 Variation of summer scPDSI in the EEP during 943–2019 CE.** (a) The reconstruction results (orange curve) are categorized into dry/wet years or extremely dry/extremely wet years based on ±1σ or ±2σ of the mean, respectively. A 10-year low-pass filter was applied to capture interdecadal fluctuations (blue curve). (b) The scPDSI kernel density probability distribution for the EEP for different climatic periods. (c) Kite diagrams and (d) cumulative point-line plots for dry, wet, or extreme dry/extreme wet events, together with a 10-year moving percentile filter, used to capture the long-term variations of cumulative events. It is evident that the EEP was generally humid over the last millennium. Whether during the LIA or MWP, the overall probability density of the scPDSI index is distributed between 0 and 2. The cumulative occurrences of extremely dry and extremely wet events during the LIA are notably lower than those during the MWP and CWP. Conversely, the MCA and MWP have a higher frequency of extreme dry and extreme wet events. This phenomenon may be influenced by temperature, specifically, increased precipitation resulting from warming; however, further research is needed to explore this relationship (Griffiths et al., 2016).


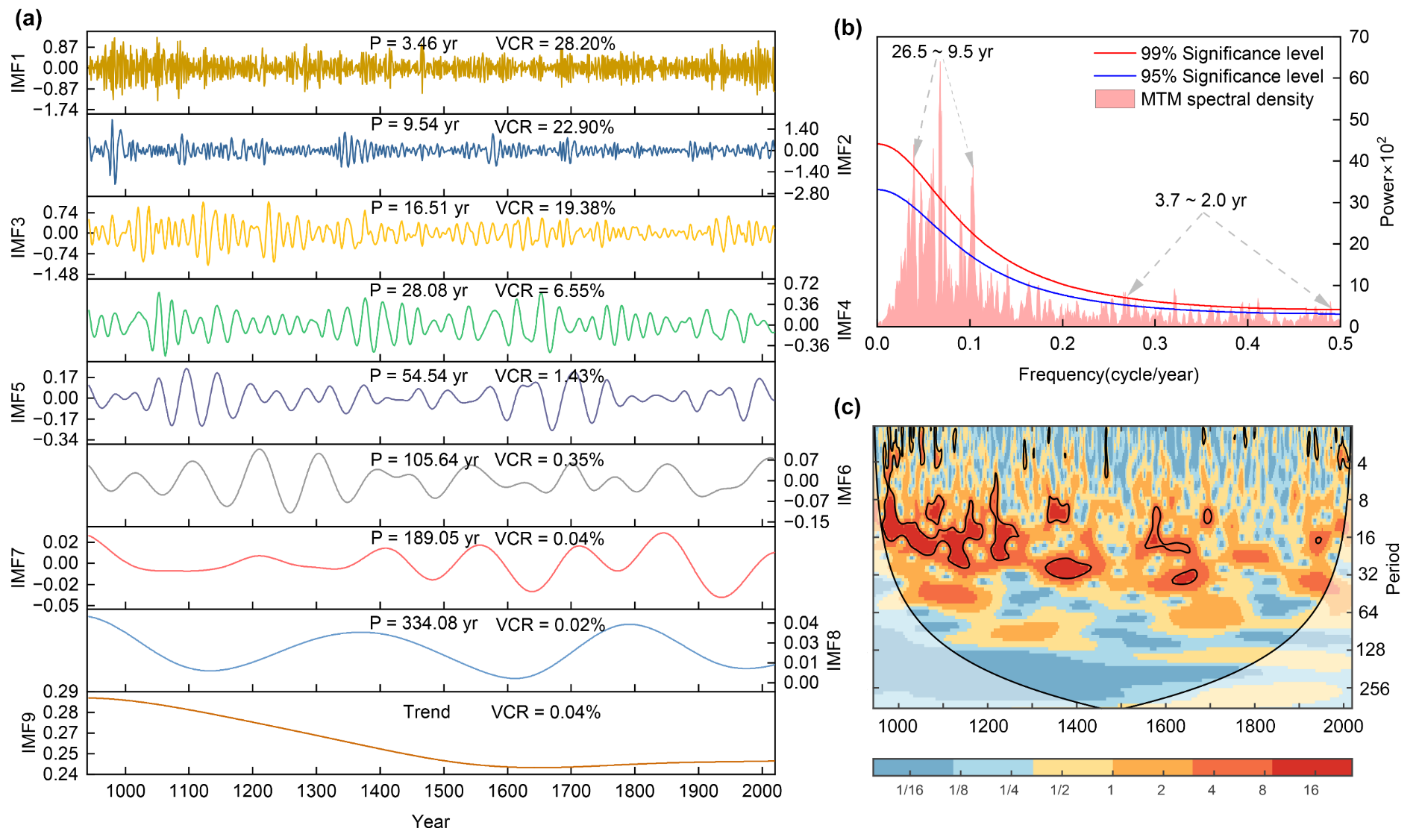


**Supplementary Fig. 8 Cyclicities within the reconstructed scPDSI of the EEP using multiple methods.** (a) EEMD results for reconstructed scPDSI, where P represents the period, VCR represents the contribution variance, and IMF represents the intrinsic mode function. (b) Spectral analysis results for the reconstructed scPDSI using the MTM. The orange fill represents spectral density, with noise tests at the 99% (red line) and 95% (blue line) significance levels. (c) Wavelet power spectrum analysis results for the reconstruction. Data that fail the 90% confidence test are indicated, and areas that pass the 95% confidence test are outlined in black. Significant cyclical fluctuations in scPDSI across the EEP over the last millennium are evident. Interdecadal periods ranging from 9.5 yr to 28.0 yr contribute significantly to overall dry and wet variations, and interannual changes within the 2.0–3.70 years range also make a substantial contribution. These results support the spatial correlation model, suggesting that scPDSI fluctuations in the EEP over the last millennium were influenced by air-sea interactions, leading to variations on interannual to interdecadal timescales. The EEP also shows a long-term trend of increasing drought.


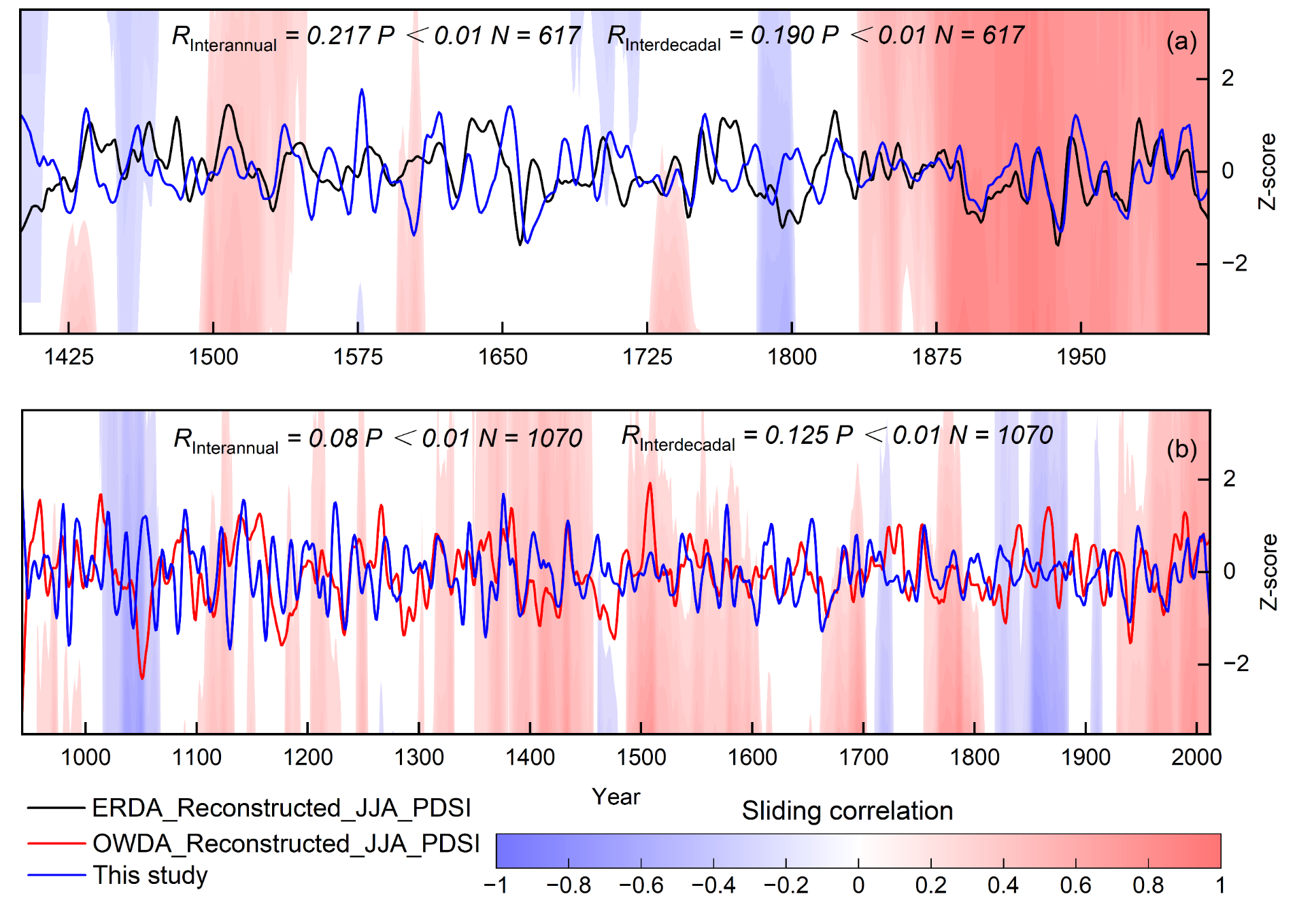


**Supplementary Fig. 9** Comparison of the scPDSI reconstruction for the EEP with (a) the European Russia Drought Atlas (ERDA) and (b) Old World Drought Atlas (OWDA) (Cook et al., 2015; Cook et al., 2020). The selected grid points span 50°N–60°N and 29.5°E–50°E, taking the common coverage time range as the selected time range, and with 10-year low-pass filtering to highlight interdecadal fluctuations. For the reconstruction results used for comparison, we use sliding correlation with a 50-year step size to analyze the high-frequency and low-frequency sequences, and use contours for interpolation filling, and the parts that fail the significance test are masked out. Note the significant positive correlation between the reconstruction and comparison results, although differences are also evident. For the ERDA there is a weak correlation before 1800, with some intervals showing negative correlations. For OWDA, discrepancies are evident over certain intervals, such as during 1800–1900 CE and 1030–1080 CE. The results emphasize the stability and reliability of the reconstruction and highlight the importance of enhancing the sample density within the dataset as the primary means of refining the climate field reconstruction.


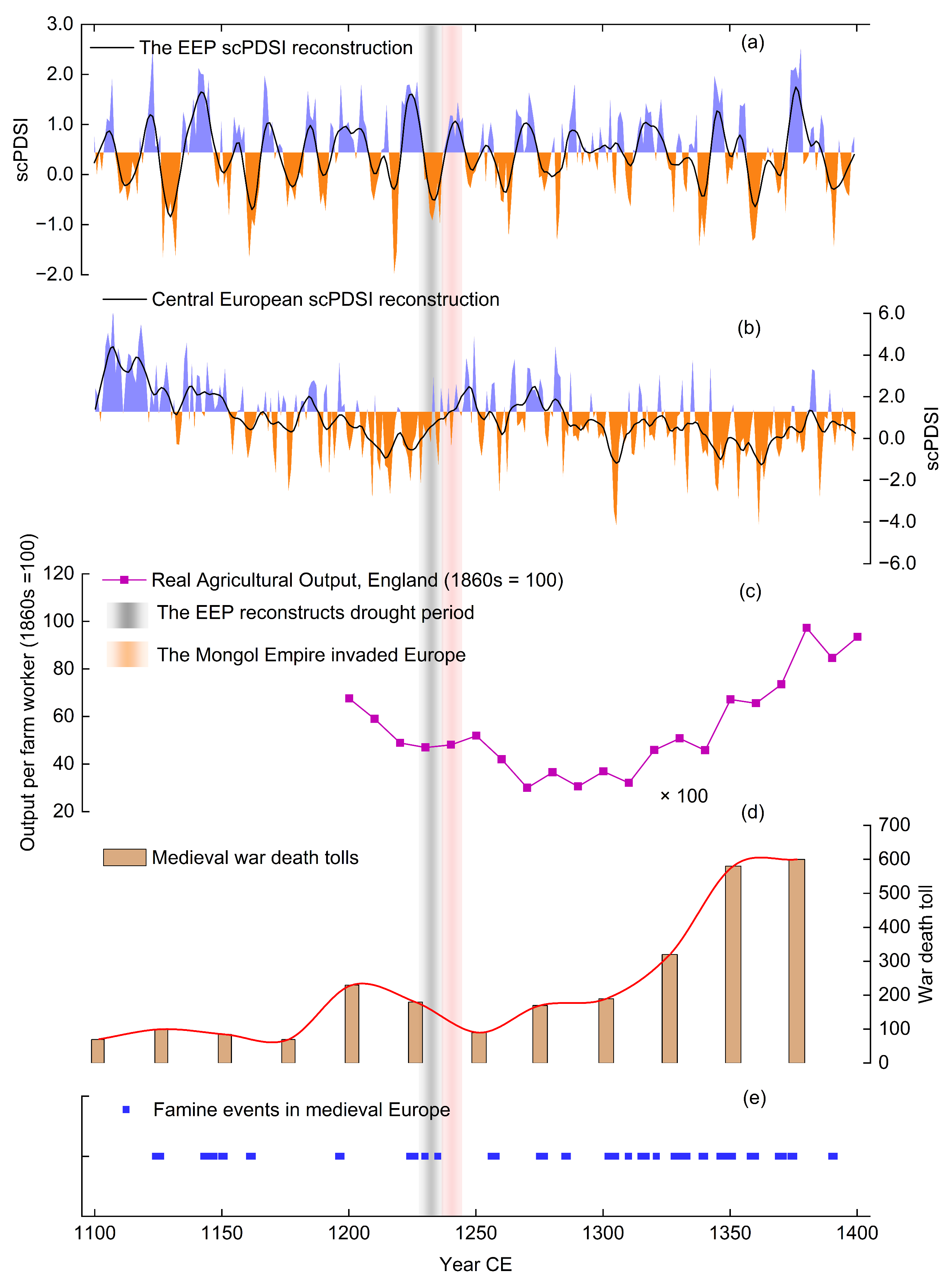


**Supplementary Fig. 10** Reconstructed wet/dry variations for 1100–1399 CE in Eastern Europe (a) and Central Europe (b), compared with war-related deaths in Europe (c), agricultural labor productivity in England (d), and the frequency of famine events in Europe. Ten year low-pass filtering is used to capture interdecadal fluctuations in the reconstruction and to categorize them into dry/wet periods based on mean values. The agricultural output for England is based on statistical data from Clark (2018). Comprehensive data on agricultural or grain prices from the early- to mid-Middle Ages (pre-1250 CE) are not readily available, and thus data for England were used as a substitute. While it may not fully represent changes across all of Europe, it provides a glimpse and perspective into socioeconomic changes. Due to the limited available literature, a precise chronology could not be established for war deaths, as the recorded death toll is an accumulation over 25 years (the data comes from the sum of 9 countries including France, Italy, Russia, Spain, the United Kingdom, Austria, the Netherlands, Poland and Germany) (Sorokin, 2017). Famine events span the geographical boundaries of modern Europe (Alfani and Gráda, 2017). The figure shows that Europe experienced major climatic and societal challenges including drought, internal conflicts, and famine prior to the conquest by the Mongol Empire, and these adverse conditions were advantageous to the Mongols. The Mongol cavalry rapidly conquered Eastern and Central Europe, and advanced to the Danube River. We suggest that while drought may have prompted the Mongols to invade Eastern Europe, it is important to recognize that the divided and turbulent state of Europe also expedited the Mongol conquest of the continent.


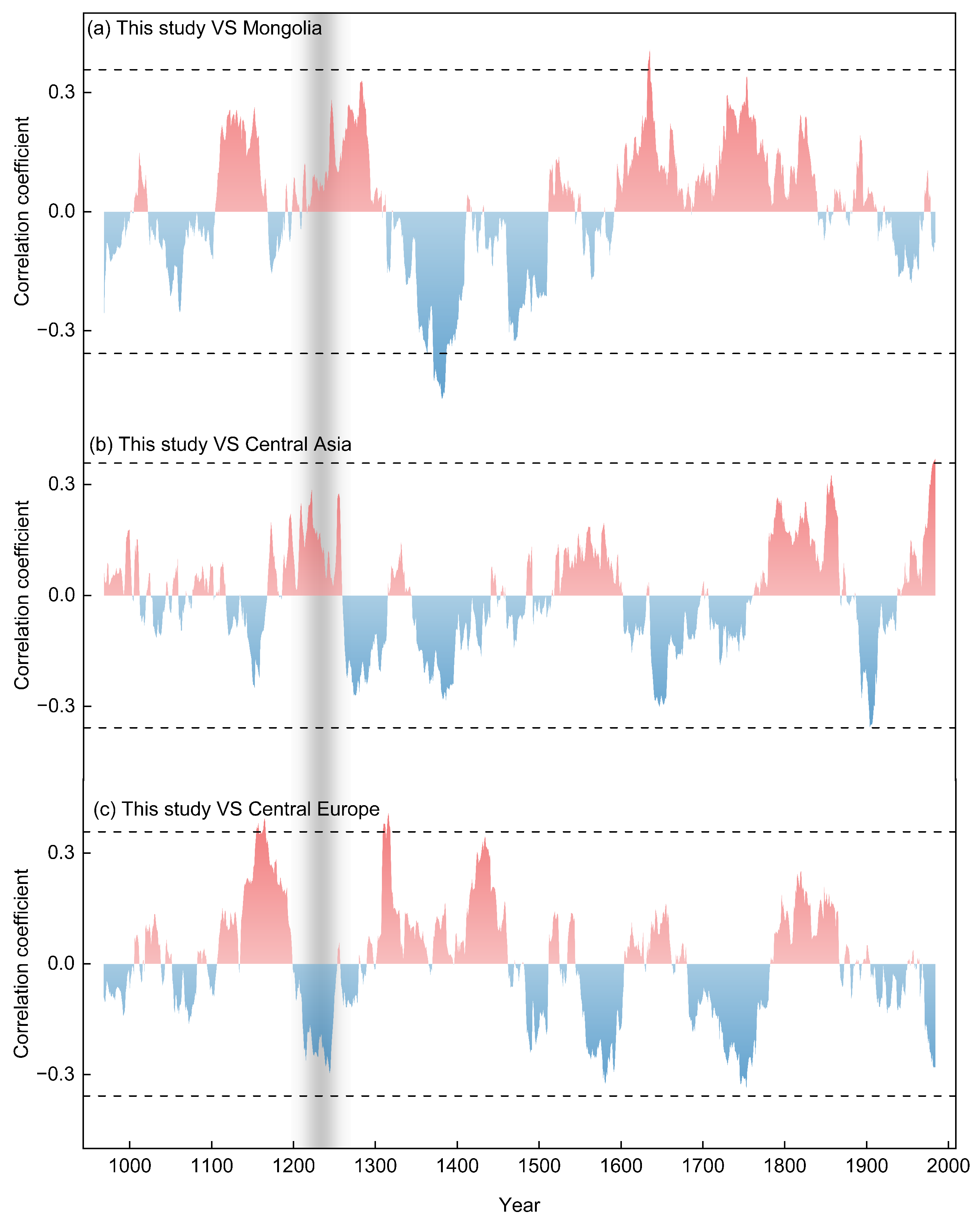


**Supplementary Fig. 11 Historical dynamic changes in water balance in the main areas of the Mongol Empire's Western Expeditions.** This study reconstructed scPDSI demonstrates dynamic relationships with (a) the reconstructed scPDSI of the MP, (b) the reconstructed precipitation in CA, and (c) the reconstructed scPDSI in Central Europe. A moving correlation analysis with a 51-year step window is used, with positive correlations filled in pink upwards and negative correlations in light blue downwards, and a 0.05 confidence level test indicated by black dashed lines. Overall, the reconstructed water balance in EEP does not show a stable dynamic relationship with the moisture reconstructions of the MP, CA, and Central Europe over the past millennium. However, from the graph, we can observe that during the primary period of the Mongol Empire's western conquest (1218–1260 CE), the water balance reconstruction in EEP maintained a positive dynamic relationship with the water reconstructions of the MP and CA, while maintaining a negative dynamic relationship with Central Europe. From this, we can infer that the long-term coordinated droughts across Eurasia facilitated the westward aggression and expansion of the Mongol Empire, while the halt in Central Europe was due to the long-term heterogeneous relationship within Europe in both space and time, leading the Mongols to cease their invasion as drought transitioned to wetness and maintained a prolonged negative correlation.


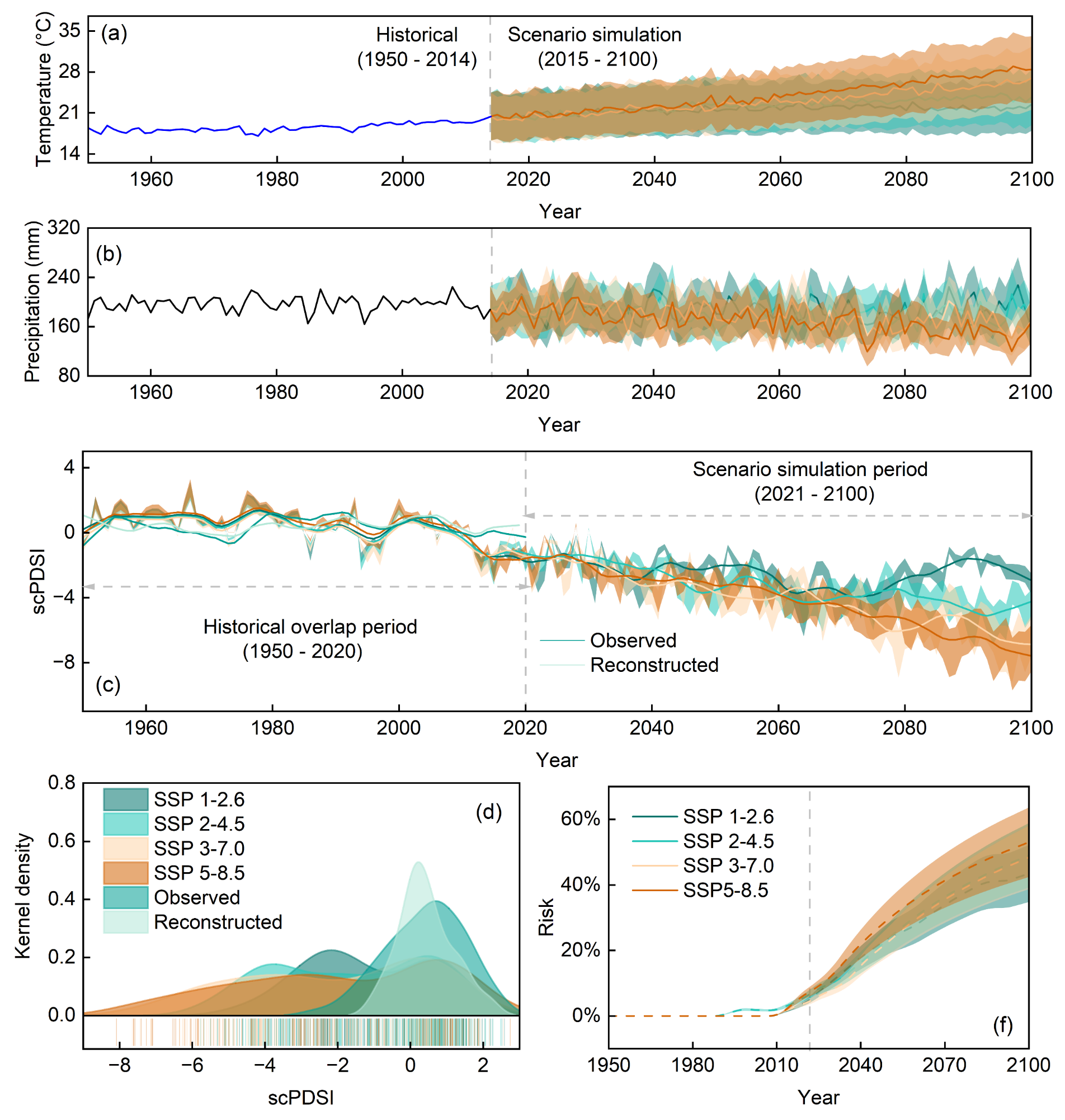


**Supplementary Fig. 12 Multi-model predictions of the scPDSI in the EEP and estimates of the risk of future extreme drought.** (a) Summer (JJA) mean temperatures from the seven CMIP6 models used in this study. Different colored lines represent various SSP, with the uncertainty interval from 20% to 80% added (shaded area). (b) Summer (JJA) total precipitation from the seven CMIP6 models used in this study. Similar to (a), different lines represent different SSPs with the same uncertainty interval. (c) Multi-model ensemble calculations of scPDSI for the EEP based on temperature and precipitation data under different SSP scenarios. Ten-year moving window low-pass filtering is used to highlight interdecadal fluctuations. The shaded area represents an uncertainty range of 20% to 80%. (d) Kernel density probability estimation analysis of the overall distribution of scPDSI in EEP, under the reconstruction, observation, and different SSP scenarios. The lower axis shows the actual data distribution. (e) The risk of extreme drought events based on scPDSI changes in the EEP under different SSP scenarios, using the method of Heeter et al. (2023). The results are calculated as the scPDSI mean minus twice the standard deviation (mean-2σ) from 1950 to 2020. The final results were smoothed using 15-year moving window low-pass filtering and includes an error interval of 20% to 80%. The CMIP6 multi-model ensemble results indicate that the EEP is likely to experience increasingly warm and dry summers over the next 80 years. In the context of different economic development paths, even under the SSP1-2.6 scenario, temperatures are 2°C higher than in 2014 by the end of this century, while the high emissions scenario shows an 8°C increase. Compared with temperature, precipitation shows differences under different SSP scenarios: from a decrease to an increase in all economic development paths after 2074. For instance, under SSP1-2.6, total precipitation levels are projected to reach 227.4 mm by the end of this century, before returning to pre-2014 mean levels, but still indicating generally dry conditions. The rapid temperature rise increases the evaporation rate, while the gradual decrease in precipitation cannot compensate for this increased drying effect. Consequently, the scPDSI effectively reflects this warming and drying trend, even under the SSP1-2.6 scenario. This leads to a persistent drought state, with the probability of extreme drought events in the EEP significantly increasing in the future. Nonetheless, the multi-model fusion data results suggest a future characterized by continued drought, but with significant uncertainties.


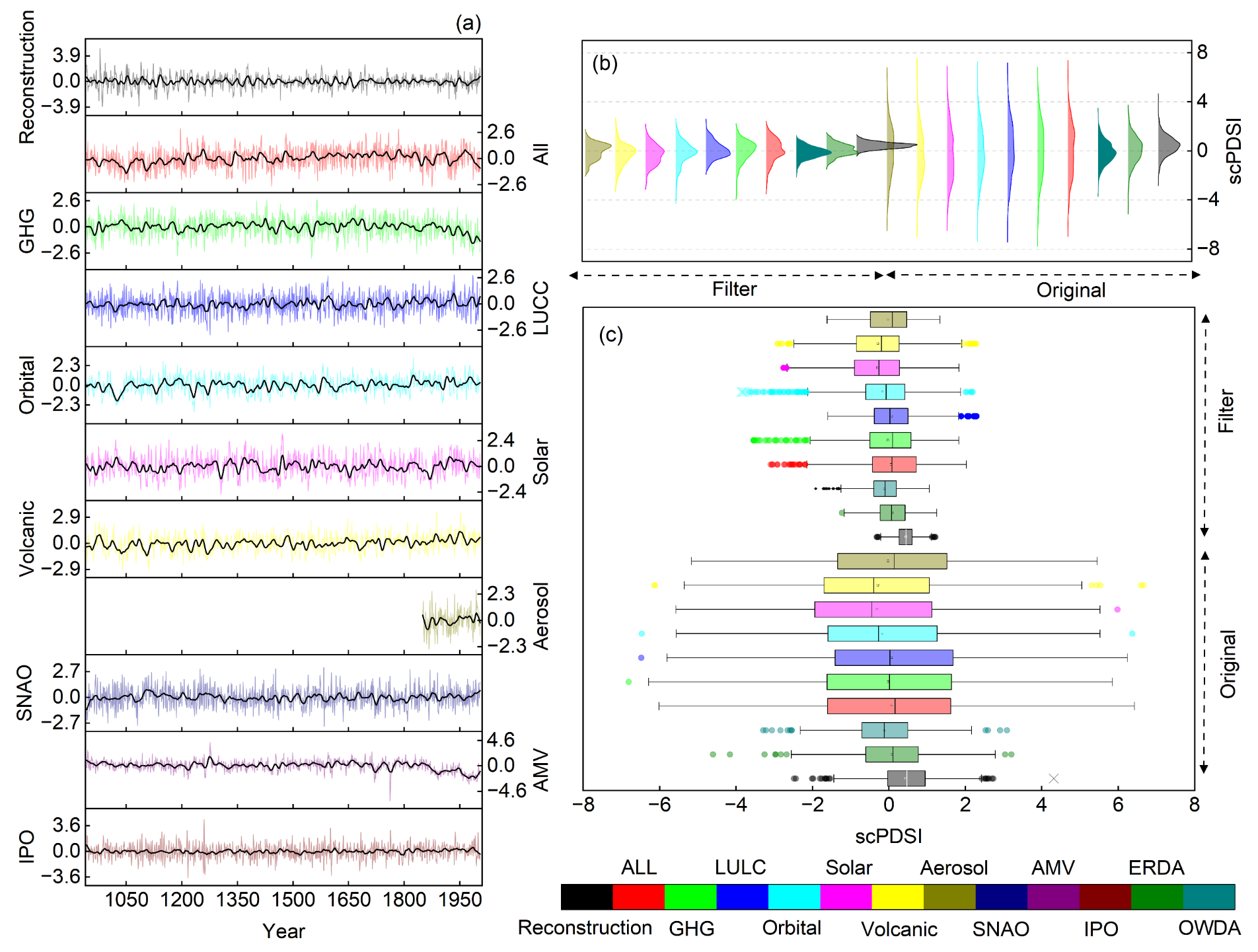


**Supplementary Fig. 13 Comparison of scPDSI calculated under various single forcing factors or all forcing factors, based on CESM-LME model data with tree-ring-reconstructed scPDSI.** (a) The scPDSI and the air-sea index under various single-forcing and full-forcing backgrounds are calculated based on CESM-LME model data.(b) Ridge plots comparing the scPDSI with different single forcings and full forcing factors and the reconstruction results. (c) Box plots comparing the scPDSI and reconstruction results for different single forcing factors and full forcing. The box represents the 25–75% interquartile range, the cap is the 1.5 IQR (interquartile range), and the symbol indicates the mean or outlier. All results are normalized as Z-scores and low-pass filtered with a 20-year window to highlight interdecadal fluctuations. The left side is the result after 20-year low-pass filtering, and the right side is the original data. The calculation of the scPDSI for various single forcings and total forcing additions in the figure is consistent with the method in the CMIP6 model prediction. The potential evapotranspiration (PET) is calculated using the Thornthwaite formula with the summer average temperature data, and then combined with the total summer precipitation data to calculate the scPDSI. The summer North Atlantic Oscillation (SNAO) is identified as the EOF1 of the sea level pressure (SLP) anomalies within the domain 30°N–85°N, 70°W–30°E. Correspondingly, standardized time series of the first principal components are taken as the SNAO index (Hong et al., 2022). The Atlantic Multidecadal Variability (AMV) is identified as the area-averaged sea surface temperature (SST) anomalies over the North Atlantic region (0°– 60°N, 75°W– 7.5°W). The standardized anomalies are taken as the AMV index (Ting et al., 2009). The Interdecadal Pacific Oscillation (IPO) is identified as the second empirical orthogonal function (EOF2) of low-pass filtered SST anomalies over the Pacific Ocean (60°S–60°N, 110°E–70°W). The standardized time series of the corresponding principal component (PC2) is taken as the IPO index (Henley et al., 2015). The definition of all indices is done in the CESM-LME model. The MLR method was used to fit the simulated scPDSI time series and air-sea indices as explanatory variables, assuming the residuals are noise. The MLR analysis shows that changes in scPDSI are caused by these known climatic factors plus noise. All regressors are normalized using Z-scores and then smoothed with a 20-year low-pass filter to highlight low-frequency signals. The scPDSI time series are fitted as follows:

${scPDSI}_{ALL}\left( t \right)= \beta_{0}+\beta_{1}SNAO\left( t \right)+\beta_{2}AMV\left( t \right)+\beta_{3}IPO\left( t \right)+\beta_{4}{scPDSI}_{GHG}\left( t \right)+\beta_{5}sc{PDSI}_{LULC}\left( t \right)+\beta_{6}{scPDSI}_{Orbital}\left( t \right)+\beta_{7}{scPDSI}_{Solar}\left( t \right)+\beta_{8}sc{PDSI}_{Volcanic}\left( t \right)+\beta_{9}{scPDSI}_{Aerosol}\left( t \right)$ (1)

where scPDSI*_i_* represents a single forcing simulations, including the GHG, LULC, Orbital, Solar, Volcanic, and Ozone/Aerosol simulations. The contribution of each explanatory variable, namely, the explained variance (EV), is calculated as follows:

${EV}_{i}=\frac{\beta_{i}R^{2}}{\sum_{i=1}^{n} \beta_{i}} \times100\%$ (2)

where *R^2^* is the square of the correlation coefficient between the scPDSI simulated by fully forced simulations and those estimated by all variables using MLR and *β*_i_ is the corresponding variable regression coefficient in Equation (1).

**Supplementary Table 1.** Details about the living-tree chronologies.

| **Site code** | **Lat, N** | **Lon, E** | **H, m** | **Species** | **No. of trees** | **No. of cores** | **First year** | **Last year** | **ITRDB code** |
| --- | --- | --- | --- | --- | --- | --- | --- | --- | --- |
| L10S | 57.3458 | 36.6186 | 155 | PISY | 12 | 24 | 1849 | 2014 | RUS344 |
| X01S | 56.5435 | 44.7969 | 108 | PISY | 18 | 33 | 1834 | 2014 | RUS383 |
| T24S | 55.9090 | 48.7333 | 86 | PISY | 15 | 29 | 1749 | 2016 | RUS374 |
| T25S | 55.9078 | 48.7323 | 79 | PISY | 15 | 27 | 1765 | 2016 | RUS375 |
| M13S | 55.7338 | 36.8408 | 167 | PISY | 20 | 35 | 1763 | 2014 | RUS347 |
| M18E | 55.6961 | 36.7280 | 199 | PCAB | 18 | 35 | 1885 | 2013 | RUS348 |
| T01S | 55.3000 | 49.2600 | 66 | PISY | 15 | 25 | 1749 | 2014 | RUS358 |
| M19S | 54.9123 | 37.6564 | 181 | PISY | 25 | 40 | 1834 | 2014 | RUS349 |
| Z1-2S | 54.7708 | 43.4049 | 180 | PISY | 21 | 39 | 1881 | 2014 | RUS386 |
| T08S | 54.4268 | 52.7574 | 240 | PISY | 18 | 31 | 1809 | 2015 | RUS365 |
| OPP | 54.0493 | 35.8307 | 154 | PISY | 12 | 22 | 1717 | 2010 | RUS351 |
| H12E | 53.9633 | 35.8131 | 166 | PCAB | 13 | 25 | 1910 | 2014 | RUS330 |
| T06S | 53.4064 | 49.9731 | 162 | PISY | 21 | 42 | 1828 | 2015 | RUS363 |
| T05S | 52.9539 | 52.0585 | 79 | PISY | 21 | 41 | 1798 | 2015 | RUS362 |
| T23S | 52.5800 | 48.0000 | 256 | PISY | 21 | 41 | 1921 | 2015 | RUS373 |
| T22S | 52.5000 | 48.0400 | 270 | PISY | 19 | 35 | 1815 | 2015 | RUS372 |
| T13S | 52.3600 | 42.6000 | 128 | PISY | 15 | 27 | 1827 | 2015 | RUS369 |
| T03S | 51.2011 | 40.1993 | 94 | PISY | 21 | 36 | 1741 | 2014 | RUS360 |
| DL | 50.7299 | 46.6695 | 60 | QURO | 16 | 29 | 1908 | 2008 | RUS328 |
| V03S | 50.6833 | 37.7960 | 100 | PISY | 14 | 22 | 1790 | 2014 | RUS380 |
| NOV | 58.5132 | 31.2765 | 90 | PISY | 42 | 45 | 1841 | 2019 | n/a |

Note: PISY is *Pinus sylvestris L.*, PCAB is *Picea abies (L.) Karst., P.obovata Lebed.*, QURO is *Quercus robur R*. Lat is latitude, Lon is longitude, H is altitude.

**Supplementary Table 2.** Details about the historical chronologies.

| **Site code** | **Lat, N** | **Lon, E** | **Species** | **No. of trees** | **No. of cores** | **First year** | **Last year** | **ITRDB code** |
| --- | --- | --- | --- | --- | --- | --- | --- | --- |
| KARELIA | 60.8 - 62.72 | 33.06 - 35.27 | PISY, PCAB | 67 | 34 | 1376 | 1767 | RUS335 |
| KIRILLOV | 59.86 - 59.86 | 38.37 - 38.37 | PISY, PCAB | 71 | 138 | 1085 | 1744 | RUS337 |
| VOLOGDA | 59.22 - 59.22 | 39.89 - 39.89 | PISY, PCAB | 105 | 206 | 1518 | 1881 | RUS381 |
| NOVGOROD | 58.52 - 58.52 | 31.27 - 31.27 | PISY, PCAB | 71 | 103 | 1149 | 1814 | RUS350 |
| KOSTROMA | 57.6 - 58.20 | 40.8 - 41.3 | PISY, PCAB | 66 | 87 | 1479 | 1804 | RUS339 |
| ZD1 | 56.04 - 56.41 | 31.97 - 32.22 | QURO | 57 | 121 | 572 | 1382 | RUS387 |
| ZD2 | 56.04 - 56.41 | 31.97 - 32.22 | QURO | 34 | 70 | 1346 | 1762 | RUS388 |
| SMOLENSK | 54.77 - 54.77 | 32.05 - 32.05 | PISY | 7 | 14 | 1387 | 1624 | RUS356 |

Note: PISY is *Pinus sylvestris L.*, PCAB is *Picea abies (L.) Karst., P.obovata Lebed.*, QURO is *Quercus robur R*. Lat is latitude, Lon is longitude, H is altitude.

**Supplementary Table 3**. The main time points of the Mongol Empire's

|  | Time point | General history |
| --- | --- | --- |
| The Rise of the Mongol Empire | 1178 – 1206 CE | Temujin was awarded the title of Genghis Khan and became the leader of the Mongol tribe. |
|  | 1207 – 1217 CE | The Mongol Empire began to expand, completing the conquest of Xi Xia and the Jin Dynasty. |
| The expansion and division of the Mongol Empire | 1218 – 1236 CE | The empire continued its westward expansion by successfully conquering Khwarezm and overcoming obstacles to further western advancement. The Battle of the Kalka River marked the inception of the empire's expansion into Europe. Simultaneously, the Mongol army crossed the New Kush Mountains and the Yellow River to accomplish the conquest of East Asia and South Asia. It's noteworthy that Genghis Khan passed away during this period, but his descendants faithfully carried on his unfinished legacy. |
|  | 1237 – 1242 CE | The Mongol Empire's invasion of the Rus Principality and Ryazan signified the Mongols' successful penetration of the eastern barriers to European invasion. Furthermore, swift battles and conquests expanded their frontiers to the Danube River Basin. Yet, why the path of western expansion came to a halt in Hungary remains a topic that sparks varied opinions. |
|  | 1243 – 1270 CE | After expanding into Eastern Europe, Central Asia, and the Middle East, the Mongol Empire fragmented into various khanates, including the Ilkhanate, the Golden Horde, the Great Mongol State, and the Chagatai Khanate. This marked the era of the Mongol Empire's division. |
| The end of the Mongol Empire | 1271 – 1368 CE | Kublai Khan assumed the title of Great Khan within the Ilkhanate and founded the Yuan Dynasty. The Yuan Dynasty reigned until the establishment of the Ming Dynasty, which marked the end of Yuan rule. Concurrently, various regimes led by Mongol descendants gradually declined and the Mongol Empire eventually crumbled, bringing it to a definitive conclusion. |

**Supplementary Table 4**. Number of ensemble members for each model and SSP scenario of the multi-model CMIP6 ensemble used to calculate future drought in the Eastern European Plain, and the references submitted to CMIP6 for each model.

| Model | SSP1-2.6 | SSP2-4.5 | SSP3-7.0 | SSP5-8.5 | ECS | Reference |
| --- | --- | --- | --- | --- | --- | --- |
| CESM2-WACCM | 1 | 1 | 1 | 1 | 4.7 | Danabasoglu, (2023a) |
| IPSL-CM6A-LR | 3 | 2 | 10 | 1 | 4.5 | Boucher et al., (2023) |
| UKESM1-0-LL | 5 | 5 | 5 | 4 | 5.3 | Tang et al., (2023) |
| CNRM-ESM2-1 | 5 | 5 | 5 | 5 | 4.8 | Voldoire, (2023) |
| CNRM-CM6-1 | 6 | 6 | 6 | 6 | 4.8 | Voldoire, (2023) |
| CESM2 | 1 | 1 | 2 | 2 | 5.2 | Danabasoglu, (2023b) |
| CanESM5 | 9 | 9 | 9 | 9 | 5.6 | Swart et al., (2019) |

Note. ECS values are taken from Pendergrass (2019) and https://www.carbonbrief.org/cmip6-the-next-generation-of-climate-models-explained.

**References**

1. Alfani, G., Gráda, C.Ó., 2017. Famines in Europe: An Overview, in: Ó Gráda, C., Alfani, G. (Eds.), Famine in European History. Cambridge University Press, Cambridge, pp. 1-24.
2. Beckers, J.M., Rixen, M., 2003. EOF Calculations and Data Filling from Incomplete Oceanographic Datasets. Journal of Atmospheric and Oceanic Technology 20, 1839-1856.
3. Biondi, F., Waikul, K., 2004. DENDROCLIM2002: A C++ program for statistical calibration of climate signals in tree-ring chronologies. Computers & Geosciences 30, 303-311.
4. Boucher, O., Denvil, S., Levavasseur, G., Cozic, A., Caubel, A., Foujols, M.-A., Meurdesoif, Y., Ghattas, J., Cadule, P., Ducharne, A., Vuichard, N., Cheruy, F., 2023. IPSL IPSL-CM6A-LR model output prepared for CMIP6 LS3MIP. World Data Center for Climate (WDCC) at DKRZ.
5. Brouder, S.M., Volenec, J.J., 2008. Impact of climate change on crop nutrient and water use efficiencies. Physiologia Plantarum 133, 705-724.
6. Büntgen, U., Trouet, V., Frank, D., Leuschner, H. H., Friedrichs, D., Luterbacher, J., & Esper, J. 2010. Tree-ring indicators of German summer drought over the last millennium. Quaternary Science Reviews, 29(7-8), 1005-1016.
7. Büntgen, U., Tegel, W., Nicolussi, K., McCormick, M., Frank, D., Trouet, V., ... & Esper, J. 2011. 2500 years of European climate variability and human susceptibility. science, 331(6017), 578-582.
8. Chanapathi, T., Thatikonda, S., Raghavan, S., 2018. Analysis of rainfall extremes and water yield of Krishna river basin under future climate scenarios. Journal of Hydrology: Regional Studies 19, 287-306.
9. Chen, F., Shang, H., Panyushkina, I.P., Meko, D.M., Yu, S., Yuan, Y., Chen, F., 2019. Tree-ring reconstruction of Lhasa River streamflow reveals 472 years of hydrologic change on southern Tibetan Plateau. Journal of Hydrology 572, 169-178.
10. Clark, G. (2018). Growth or stagnation? Farming in England, 1200–1800. The Economic History Review, 71(1), 55-81.
11. Cook, E.R., 1986. Users manual for program ARSTAN. Tree-ring chronologies of western North America: California, eastern Oregon and northern Great Basin.
12. Cook, E.R., Buckley, B.M., D'Arrigo, R.D., Peterson, M.J., 2000. Warm-season temperatures since 1600 BC reconstructed from Tasmanian tree rings and their relationship to large-scale sea surface temperature anomalies. Climate Dynamics 16, 79-91.
13. Cook, E.R., Kairiukstis, L.A., 2013. Methods of Dendrochronology: Applications in the Environmental Sciences. Springer Netherlands.
14. Cook, E.R., Seager, R., Kushnir, Y., Briffa, K.R., Büntgen, U., Frank, D., Krusic, P.J., Tegel, W., van der Schrier, G., Andreu-Hayles, L., Baillie, M., Baittinger, C., Bleicher, N., Bonde, N., Brown, D., Carrer, M., Cooper, R., Čufar, K., Dittmar, C., Esper, J., Griggs, C., Gunnarson, B., Günther, B., Gutierrez, E., Haneca, K., Helama, S., Herzig, F., Heussner, K.-U., Hofmann, J., Janda, P., Kontic, R., Köse, N., Kyncl, T., Levanič, T., Linderholm, H., Manning, S., Melvin, T.M., Miles, D., Neuwirth, B., Nicolussi, K., Nola, P., Panayotov, M., Popa, I., Rothe, A., Seftigen, K., Seim, A., Svarva, H., Svoboda, M., Thun, T., Timonen, M., Touchan, R., Trotsiuk, V., Trouet, V., Walder, F., Ważny, T., Wilson, R., Zang, C., 2015. Old World megadroughts and pluvials during the Common Era. Science Advances 1, e1500561.
15. Cook, E.R., Solomina, O., Matskovsky, V., Cook, B.I., Agafonov, L., Berdnikova, A., Dolgova, E., Karpukhin, A., Knysh, N., Kulakova, M., Kuznetsova, V., Kyncl, T., Kyncl, J., Maximova, O., Panyushkina, I., Seim, A., Tishin, D., Ważny, T., Yermokhin, M., 2020. The European Russia Drought Atlas (1400–2016 CE). Climate Dynamics 54, 2317-2335.
16. D'Arrigo, R., Wilson, R., Liepert, B., Cherubini, P., 2008. On the ‘Divergence Problem’ in Northern Forests: A review of the tree-ring evidence and possible causes. Global and Planetary Change 60, 289-305.
17. Danabasoglu, G., 2023a. IPCC DDC: NCAR CESM2-WACCM model output prepared for CMIP6 CMIP. World Data Center for Climate (WDCC) at DKRZ.
18. Danabasoglu, G., 2023b. NCAR CESM2 model output prepared for CMIP6 LUMIP. World Data Center for Climate (WDCC) at DKRZ.
19. Griffiths, M.L., Kimbrough, A.K., Gagan, M.K., Drysdale, R.N., Cole, J.E., Johnson, K.R., Zhao, J.-X., Cook, B.I., Hellstrom, J.C., Hantoro, W.S., 2016. Western Pacific hydroclimate linked to global climate variability over the past two millennia. Nature Communications 7, 11719.
20. Friedrichs, D. A., Büntgen, U., Frank, D. C., Esper, J., Neuwirth, B., & Löffler, J. 2009. Complex climate controls on 20th century oak growth in Central-West Germany. Tree physiology, 29(1), 39-51.
21. Grissino-Mayer, H.D., 2001. Evaluating crossdating accuracy: a manual and tutorial for the computer program COFECHA.
22. Haneca, K., Čufar, K., & Beeckman, H. 2009. Oaks, tree-rings and wooden cultural heritage: a review of the main characteristics and applications of oak dendrochronology in Europe. Journal of Archaeological Science, 36(1), 1-11.
23. Heeter, K.J., Harley, G.L., Abatzoglou, J.T., Anchukaitis, K.J., Cook, E.R., Coulthard, B.L., Dye, L.A., Homfeld, I.K., 2023. Unprecedented 21st century heat across the Pacific Northwest of North America. npj Climate and Atmospheric Science 6, 5.
24. Henley, B. J., Gergis, J., Karoly, D. J., Power, S., Kennedy, J., & Folland, C. K. (2015). A tripole index for the interdecadal Pacific oscillation. Climate dynamics, 45(11), 3077-3090.
25. Hersbach, H., Bell, B., Berrisford, P., Hirahara, S., Horányi, A., Muñoz-Sabater, J., Nicolas, J., Peubey, C., Radu, R., Schepers, D., Simmons, A., Soci, C., Abdalla, S., Abellan, X., Balsamo, G., Bechtold, P., Biavati, G., Bidlot, J., Bonavita, M., De Chiara, G., Dahlgren, P., Dee, D., Diamantakis, M., Dragani, R., Flemming, J., Forbes, R., Fuentes, M., Geer, A., Haimberger, L., Healy, S., Hogan, R.J., Hólm, E., Janisková, M., Keeley, S., Laloyaux, P., Lopez, P., Lupu, C., Radnoti, G., de Rosnay, P., Rozum, I., Vamborg, F., Villaume, S., Thépaut, J.-N., 2020. The ERA5 global reanalysis. Quarterly Journal of the Royal Meteorological Society 146, 1999-2049.
26. Hong, X., Lu, R., Chen, S., & Li, S. (2022). The relationship between the North Atlantic Oscillation and the Silk Road pattern in summer. Journal of Climate, 35(20), 6691-6702.
27. Kottek, M., Grieser, J., Beck, C., Rudolf, B., Rubel, F., 2006. World map of the Köppen-Geiger climate classification updated.
28. Manning, S.W., 2023. Dendrochronology and Archaeology, Handbook of Archaeological Sciences, pp. 37-68.
29. Schneider, U., Becker, A., Finger, P., Meyer-Christoffer, A., Rudolf, B., Ziese, M., 2016. GPCC Full Data Reanalysis Version 7.0: Monthly Land-Surface Precipitation from Rain Gauges built on GTS based and Historic Data. Research Data Archive at the National Center for Atmospheric Research, Computational and Information Systems Laboratory, Boulder, CO.
30. Skomarkova, M.V., Vaganov, E.A., Mund, M., Knohl, A., Linke, P., Boerner, A., Schulze, E.D., 2006. Inter-annual and seasonal variability of radial growth, wood density and carbon isotope ratios in tree rings of beech (Fagus sylvatica) growing in Germany and Italy. Trees 20, 571-586.
31. Sorokin, P., 2017. Social and Cultural Dynamics: A Study of Change in Major Systems of Art, Truth, Ethics, Law and Social Relationships. Taylor & Francis.
32. Swart, N.C., Cole, J.N.S., Kharin, V.V., Lazare, M., Scinocca, J.F., Gillett, N.P., Anstey, J., Arora, V., Christian, J.R., Hanna, S., Jiao, Y., Lee, W.G., Majaess, F., Saenko, O.A., Seiler, C., Seinen, C., Shao, A., Sigmond, M., Solheim, L., von Salzen, K., Yang, D., Winter, B., 2019. The Canadian Earth System Model version 5 (CanESM5.0.3). Geosci. Model Dev. 12, 4823-4873.
33. Tang, Y., Rumbold, S., Ellis, R., Kelley, D., Mulcahy, J., Sellar, A., Walton, J., Jones, C., 2023. MOHC UKESM1.0-LL model output prepared for CMIP6 CMIP. World Data Center for Climate (WDCC) at DKRZ.
34. Ting, M., Kushnir, Y., Seager, R., & Li, C. (2009). Forced and internal twentieth-century SST trends in the North Atlantic. Journal of Climate, 22(6), 1469-1481.
35. Voldoire, A., 2023. CNRM-CERFACS CNRM-ESM2-1 model output prepared for CMIP6 ScenarioMIP ssp245. World Data Center for Climate (WDCC) at DKRZ.
36. Wang, H., Chen, Y., Pan, Y., Li, W., 2015. Spatial and temporal variability of drought in the arid region of China and its relationships to teleconnection indices. Journal of Hydrology 523, 283-296.
37. Yue, W., Chen, F., Davi, N.K., Zhang, H., Chen, Y., Zhao, X., Gao, Z., 2023. Little Ice Age cooling in the Western Hengduan Mountains, China: a 600-year warm-season temperature reconstruction from tree rings. Climate Dynamics.
